# Hot-spot empowered gold nanoparticles for theranostics in breast cancer

**DOI:** 10.7150/thno.119678

**Published:** 2025-08-30

**Authors:** Meng Tian, Shiqi Hu, Wen Sun, Ying Hu, Xingyi Ma

**Affiliations:** 1 School of Biomedical Engineering & School of Science, Harbin Institute of Technology, Shenzhen, 518055, China.; 2 Shenzhen Keyto Fluid Technology Co., Shenzhen, 518100, China.; 3 Centre for Translational Medicine, Shenzhen Bao'an Chinese Medicine Hospital, Guangzhou University of Chinese Medicine, Shenzhen, 518108, China.; 4 School of Life Science and Technology, Harbin Institute of Technology, Harbin, 150080, China.; 5 Key Laboratory of Science and Engineering for the Multi-modal Prevention and Control of Major Chronic Diseases, Ministry of Industry and Information Technology, HIT Zhengzhou Research Institute, Zhengzhou, 450000, China.; 6 Biosen International and Briteley Institute of Life Sciences, Shandong, 264600, China.

**Keywords:** gold nanoparticles, hot-spot, breast cancer, theranostics, nanostructural design

## Abstract

Breast cancer, being the most prevalent malignant tumor among women, confronts severe challenges in its early detection and precise treatment. Traditional diagnostic approaches have drawbacks in terms of sensitivity and specificity, and are invasive, thereby making it arduous to satisfy practical demands. Nanomedicine has introduced novel diagnostic and therapeutic modalities for breast cancer, particularly gold nanoparticles (AuNPs), which have been utilized on account of their distinctive optical and physicochemical attributes. Hot-spot empowered AuNPs have rapidly emerged and demonstrated significant potential in the diagnosis and treatment of breast cancer. From a technical standpoint, the design and synthesis of AuNPs hot-spot are constantly evolving, ranging from the control of number to the control of structure and efficient control of hot-spot utilizations, establishing a development model of “number-structure-efficient utilization”. Hot-spot empowered AuNPs are extensively employed in diagnosis and therapy, facilitating targeted drug delivery, photothermal and photodynamic therapy, and multimodal integration therapy, and also achieving the function of theranostics in an innovative manner. We have deliberated on the challenges and future development prospects of precision medicine for the diagnosis of early breast cancer and individualized treatment.

## 1. Introduction

**Figure 1:**
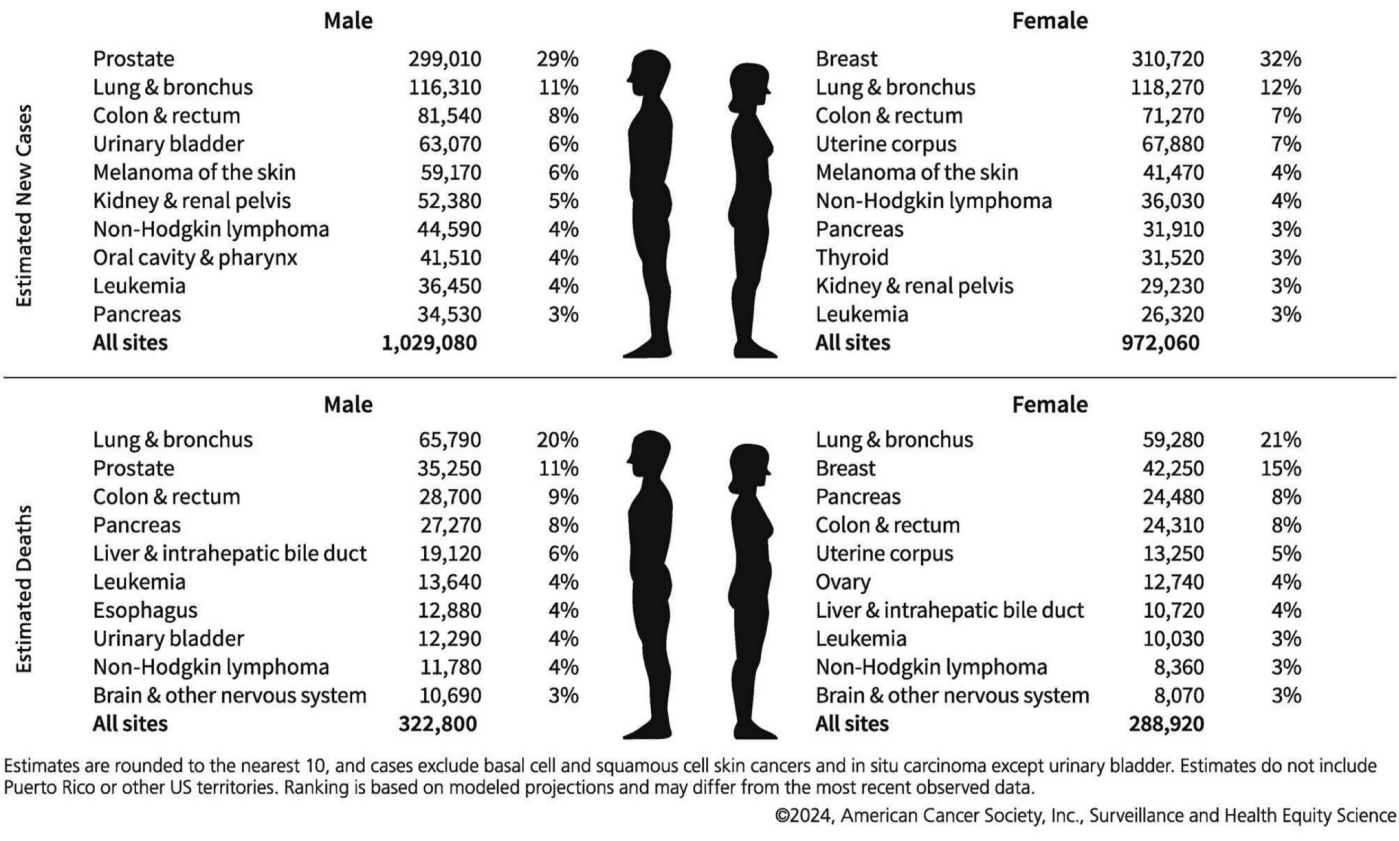
Ten leading cancer types for the estimated new cancer cases and deaths by sex, United States, 2024. Adapted with permission from 3, Copyright 2024 Wiley.

Data from the International Agency for Research on Cancer (IARC) of the World Health Organization reveals that breast cancer was the most prevalent cancer among women globally in 2020, accounting for 11.7% of all new cancer cases, with a compound annual growth rate exceeding 3% 1,2. **Figure 1** depicts the cancer types that are expected to have the highest incidence of new cases in 2024 3, thereby posing a significant threat to women's health. Breast cancers are classified into estrogenic and non-estrogenic types based on the existence of hormone receptors, namely estrogen receptor (ER), progesterone receptor (PR), and human epidermal growth factor receptor-2 (HER-2). Distinct molecular subtypes of breast cancer display disparate biological characteristics and therapeutic responses. Despite the implementation of tailored treatment protocols for other types of breast cancer, precision medicine has not yet attained the anticipated outcomes 4—targeted therapy and prioritized assessment. Genetic testing plays a crucial role in molecular diagnosis, evaluating genetic risk, monitoring treatment efficacy, identifying medication resistance, and formulating treatment plans for breast cancer, thereby significantly promoting precision medicine.

Breast cancer genetic testing is aimed at identifying nucleotide alterations in genes and evaluate their biological implications. Genetic testing typically depends on amplification technologies, and the sample preparation process can be quite time-consuming. Furthermore, conventional breast cancer genetic testing frequently necessitates tissue biopsies or the collection of substantial blood samples for analysis. Such procedures are not only operationally intricate but may also subject participants to potential trauma and discomfort.

The advancement of nanotechnology has facilitated genetic testing to detect and address mutations without direct sequencing. This approach is designed to meet the requirements of high sensitivity, wide detection range, minimal time consumption, patient-friendliness, and dynamic monitoring capabilities, while highlighting its usability for non-experts. The integration of nanomaterials with modern medicine has given rise to cross-disciplines such as nanobiomedicine, which encompasses applications in the diagnosis, monitoring, and treatment of diseases. Since its advent, nanomedicine has extensively utilized its remarkable physicochemical and structural properties for the treatment of various diseases 5-9, especially in the efficient delivery of antitumor drugs, diagnosis, and imaging 10.

Nanomaterials, such as gold nanoparticles (AuNPs) and graphene quantum dots, possess dimensions spanning from 1 to 100 nm, which are closely in line with the scale of biomolecules like DNA and proteins, thereby facilitating precise interactions with biological systems. The distinctive optical and electrical characteristics of nanotechnology enable the detection of minimal concentrations of breast cancer markers, such as the HER2 protein. This technology is capable of identifying trace signals in the early phases of cancer, facilitating early detection and enhancing cure rates. Nanomaterials are capable of integrating diverse treatment modalities, such as targeted drug delivery, photothermal therapy (PTT), photodynamic therapy (PDT), to enhance the management and optimize the treatment outcomes for patients with breast cancer. Nanomaterials demonstrate high sensitivity, specificity, precise targeting, multifunctionality, and low side effects, thereby presenting substantial advantages in the diagnosis and treatment of breast cancer.

AuNPs exhibit a remarkable localized surface plasmon resonance (LSPR) effect, which induces collective oscillations of surface electrons upon light exposure. This phenomenon enhances both the absorption and scattering of light. Consequently, AuNPs are highly suitable for applications in biosensors, fluorescence imaging, surface-enhanced Raman spectroscopy (SERS), and various optical diagnostic methods 11. The LSPR technique, when combined with AuNPs, demonstrates superior performance in detecting microscopic markers of breast cancer at early stages compared to traditional approaches such as enzyme linked immunosorbent assay (ELISA), thereby facilitating early detection and improving cure rates 12. The surface of AuNPs can be easily modified with a variety of biomolecules. Furthermore, AuNPs display excellent biocompatibility; the *in vivo* toxicity thereof can be minimized by means of surface modification techniques like Polyethylene Glycol modification (PEGylation). Compared with other inorganic nanomaterials-such as molybdenum disulfide (MoS₂) with its narrow-bandgap optical response 13 or black phosphorus (BP) known for high carrier mobility 14-AuNPs offer three unique advantages for breast cancer theranostics. (1) Tunable LSPR peaks (520-1100 nm) that match biological transparency windows, enabling deeper tissue penetration. (2) Superior surface chemistry supporting>5-fold higher biomolecule grafting density than semiconductor nanomaterials. (3) Proven biocompatibility through PEGylation strategies and renal clearance pathways, minimizing long-term accumulation risks 15. This feature facilitates metabolic elimination through the kidneys or liver while minimizing the risk of prolonged retention 16. It is expected that AuNPs will play a crucial role in precision medicine for breast cancer by enabling early detection and effective management of the disease.

Conventional approaches require high-energy lasers to effectively stimulate the nanoplasma properties on the surface of AuNPs. Nevertheless, the heat generated by these lasers might inflict damage upon biomolecules or interfere with the binding process of target molecules, potentially exerting an impact on cancer diagnosis 17. Under this backdrop, the hot-spot effect of AuNPs becomes prominent due to its distinctive electromagnetic field enhancement properties-it can conspicuously amplify the plasmon resonance signal on the surface of nanoparticles under low-energy laser or even visible light circumstances. The hot-spot effect not only remarkably boosts detection sensitivity but also alleviates the demand for high-intensity laser excitation due to its potent electromagnetic field confinement. While localized heating may occur within nanogaps, the overall thermal impact on biomolecules is substantially minimized. This is primarily attributed to the ability of hot-spot to achieve strong signal enhancement under low-power excitation conditions, thereby reducing the total energy input 18. Furthermore, the excellent thermal conductivity of AuNPs facilitates rapid dissipation of localized heat, preventing temperature accumulation in surrounding regions. This spatial confinement and efficient thermal management help preserve molecular integrity and mitigate nonspecific photothermal damage during detection 19,20.

Hot-spot are highly localized areas with pronounced local field enhancement resulting from LSPR. This phenomenon typically takes place at the tip, edge, or gap between two nanoparticles. When two nanoparticles aggregate, owing to the dipole-dipole coupling effect, the peak position of the extinction spectrum might change, typically leading to a red shift of the extinction spectrum. Quantitatively speaking, hot-spot are delineated as nanoscale regions (e.g., sub-10-nm gaps or sharp tips) in which the local electromagnetic field enhancement factor (EF) surpasses 10⁴. This value of 10⁴ serves as a threshold for notable plasmonic effects in SERS. The EF can be estimated via two principal approaches, contingent upon the application context.

For near-field plasmonic simulations, such as finite-difference time-domain (FDTD) models, EF is typically approximated using the electric field amplitude ratio:



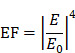



where *E* is the local electric field at the hot-spot and *E_0_* is the incident field strength. This approximation reflects the fourth-power dependence of Raman scattering intensity on the local electric field 23.

In the context of experimental characterization via SERS, the EF is defined by comparing the intensities and molecular populations in enhanced and non-enhanced environments:



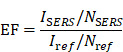



where *I_SERS_* and *I_ref_​* are the Raman intensities under enhanced and reference conditions, and *N_SERS_* and *N_ref_​* are the estimated numbers of molecules contributing to each signal, respectively. Here, *N_ref_​* is usually determined by the concentration of the analyte in the bulk solution and the area of the laser spot, while *N_SERS_* is estimated based on the surface coverage of the nanoparticles 24.

**Figure 2:**
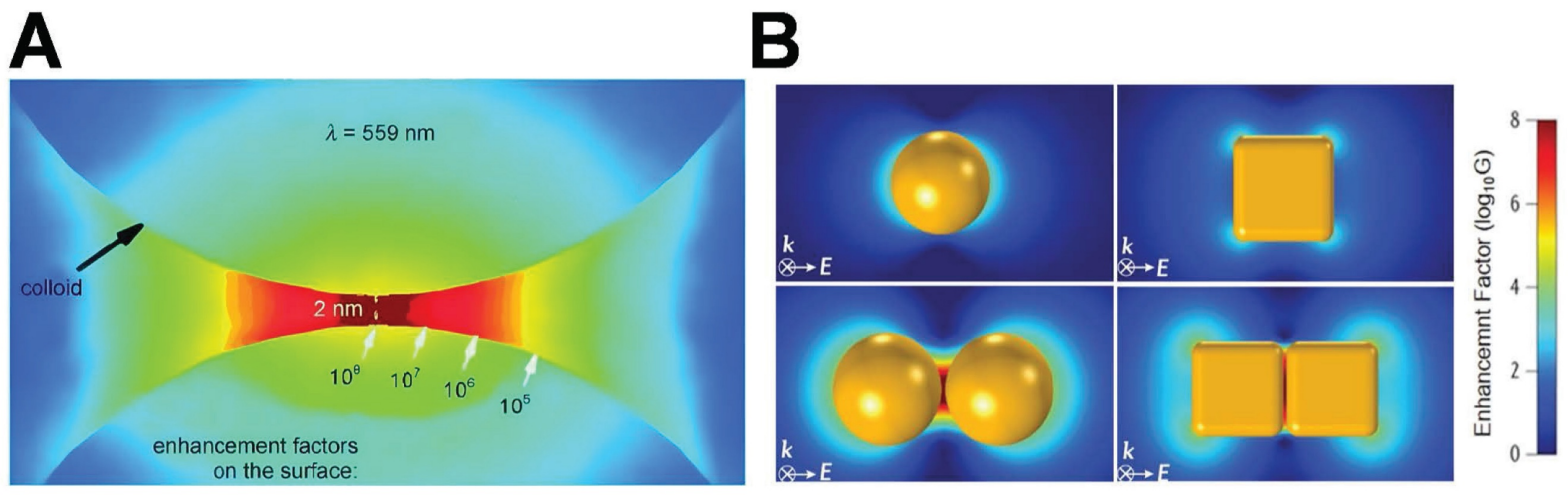
Schematic diagram of hot-spot distribution: (A) The formation of hot-spot within the gap of a nanoparticle dimer, demonstrating the exponential correlation between the interparticle distance and the EF. Adapted with permission from 21, Copyright 2010 Wiley. (B) Finite-element simulations of electric field enhancement (|E/E₀|²) for four AuNP configurations, with corresponding average/maximum EFs labeled. Excitation wavelengths: 545 nm (sphere), 585 nm (cube), 645 nm (sphere dimer), 725 nm (cube dimer). Adapted with permission from 22, Copyright 2017 American Chemical Society.

In typical hot-spot configurations, such as AuNPs dimers featuring sub-2 nm gaps or nanostars with sharp protrusions, the EF values can span from 10^4^ to 10^9^. As depicted in **Figure 2A** 21, with the thermal reduction of the spot distance, the signal will possess a large enhancement coefficient. In contrast to isolated particles, the shift of the peak position wavelength is dependent on the distance of the nanoparticles. However, aside from the prescribed interparticle distance, the shape of the particles also exerts a significant role in signal enhancement (**Figure 2B**) 22. The simulation is grounded in the Finite-element simulations. In a single spherical nanoparticle structure (characterized by a diameter of 60 nm and an excitation wavelength of 545 nm), the peak value of the EF reaches merely 2.5×10³. Conversely, for the corresponding nanocube, the maximum EF under 585 nm excitation does not surpass 2.7×10³. In contrast, when a spherical dimer structure with a 2 nm gap is fabricated and excited at 645 nm, the local EF can be elevated to 4.11×10⁹, and the average EF amounts to 1.47×10⁶, manifesting an enhancement effect of approximately six orders of magnitude. Similarly, the maximum EF of the cube dimer structure under 725 nm excitation ascends to as high as 1.24×10⁸, and the average value is 2.41×10⁵. These findings comprehensively illustrate the decisive impact of the geometric morphology of nanostructures (notably the gap size) on the local electric field enhancement capacity.

The traditional diagnostic approaches for breast cancer are beset with issues such as poor sensitivity, considerable invasiveness, and complex operation. The traditional treatment modalities are accompanied by challenges like substantial side effects and the lack of targeting. Traditional diagnostic methods for breast cancer predominantly encompass tissue biopsy 25, immunohistochemistry (IHC), fluorescence *in situ* hybridization (FISH), real-time quantitative polymerase chain reaction (qPCR), and next-generation sequencing (NGS) 26-28. Among these, tissue biopsy is an invasive approach that can induce pain or complications. Additionally, issues related to sample heterogeneity exist 29. FISH and IHC exhibit a certain level of specificity; however, they impose stringent requirements on operating conditions, possess relatively limited sensitivity, and are not conducive to real-time monitoring 30. Although qPCR and NGS provide high resolution, their complex workflows (e.g., 4-6 hours for qPCR; 3-7 days for NGS) and high cost hinder widespread use in rapid screening 31,32. These limitations underscore the need for novel platforms that combine minimal invasiveness, single-molecule sensitivity, and real-time monitoring capabilities.

**Figure 3:**
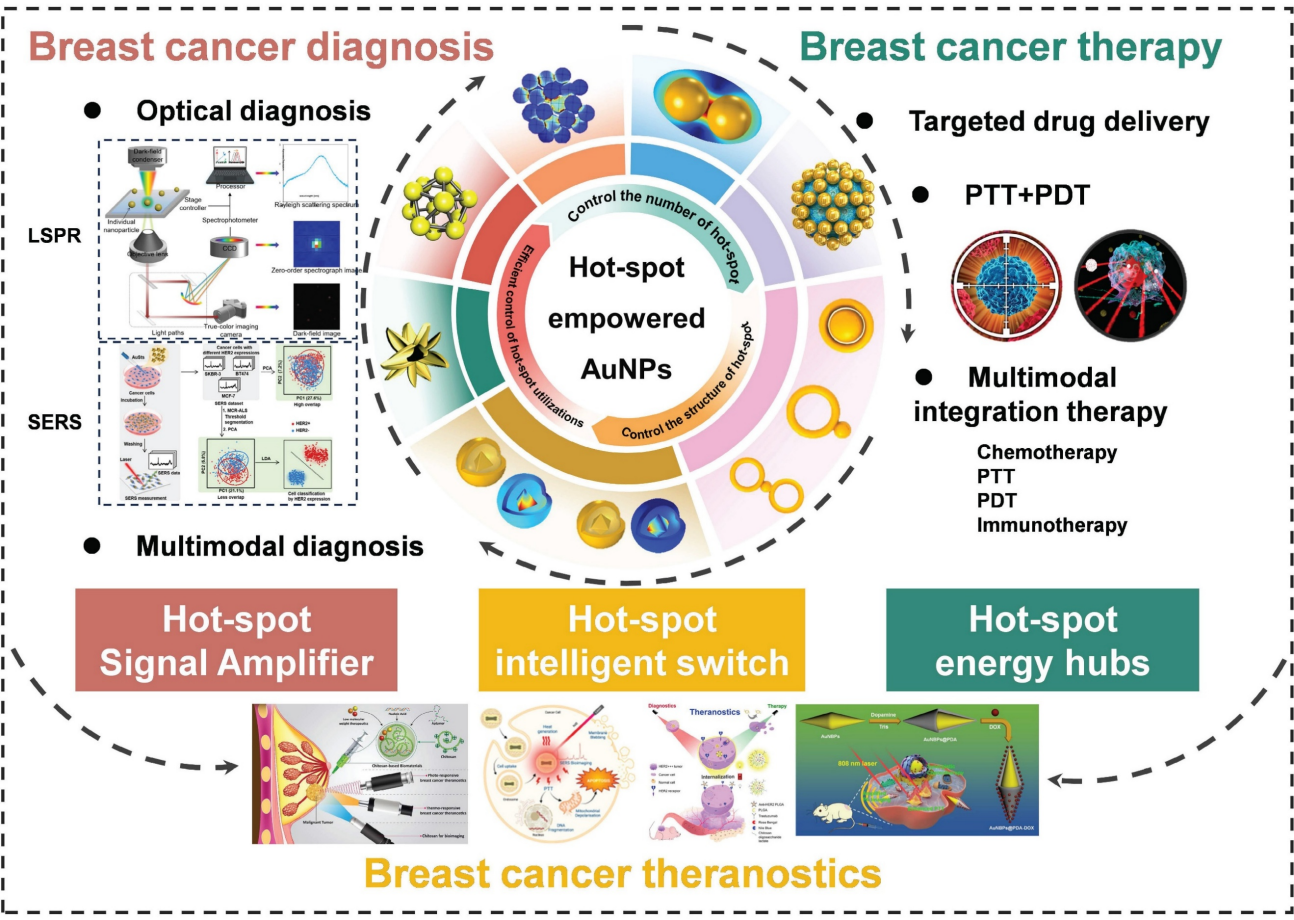
The overview of the manuscript content, highlights the synthesis strategy of hot-spot empowered AuNPs and its application in the theranostics of breast cancer.

AuNPs, featuring their unique LSPR effect, excellent biocompatibility and modifiability, demonstrate remarkable advantages in the early diagnosis and precise therapy of breast cancer. Hot-spot, being nano-regions with conspicuously enhanced electromagnetic fields on the surface of AuNPs, can significantly enhance the optical signal and energy conversion efficiency. Nevertheless, the preparation and application of them are lacking in systematic induction and summary. This review pioneered the proposition of the evolution path from the preparation to the application of hot-spot empowered AuNPs for the diagnosis and therapy of breast cancer and constructed the “number-structure-efficient utilization” development model. It not only systematically sorted out the evolution process of hot-spot technologies but also highlighted the transformation from “extensive enhancement” to “precise application”, and elaborately compared the mechanisms, advantages, and challenges at each stage. We conducted a comprehensive and systematic exploration on the application of hot-spot empowered AuNPs in the theranostics of breast cancer. By concentrating on the integrated strategy of theranostics, we filled the gap of disconnection between diagnosis and therapy in the existing studies and offered new ideas and technical frameworks for the development of precision medicine in breast cancer. In previous reviews, technical problems and potential development paths of hot-spot empowered AuNPs in the application of breast cancer have been hardly covered. To provide a more comprehensive perspective, this review will elaborate in detail the challenges encountered by hot-spot empowered AuNPs in clinical applications, and propose potential research fields, providing key theoretical support and practical guidance for promoting the transformation of AuNPs from laboratory research to clinical application (**Figure 3**).

## 2. Design and synthesis of hot-spot empowered AuNPs

### 2.1 Control the number of hot-spot

The coupling enhancement effect of LSPR emerges when two nanoparticles are in close proximity, leading to a remarkable amplification of the local electric field owing to the interaction of their surface plasmon resonances. The preliminary research was centered on synthesizing a large number of randomly distributed hot-spot to achieve the superposition of the enhancement effect. This stage emphasizes the design and assembly methods for the preparation of additional hot-spot to enhance signal strength, with the aim of maximizing the total number of hot-spot. Self-assembly of nanoparticles, template-making, and chemical reduction are several approaches utilized to arrange AuNPs into structures capable of generating numerous hot-spot, such as nanogaps or clusters. The objective is to optimize the quantity of hot-spot through the design and assembly of nanoparticles, with the aim of enhancing signal intensity 2,33,34.

**Figure 4:**
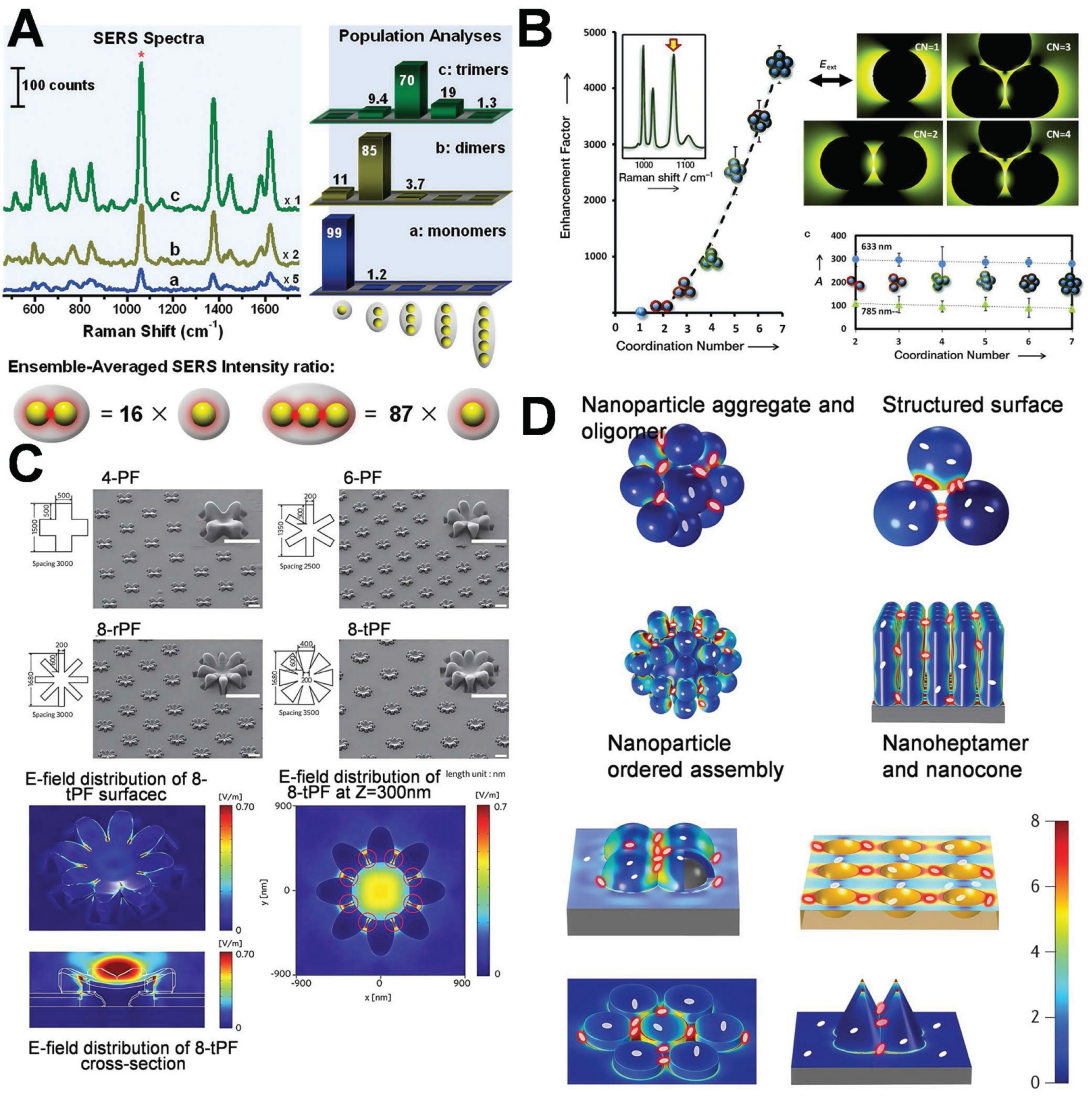
Schematic diagram of controlling the number of hot-spot: (A) SERS spectra and SERS intensity ratios of spatially isolated NPs monomer, dimer and trimer samples. Adapted with permission from 35, Copyright 2010 American Chemical Society. (B) Optical enhancement effect of nanoparticle clusters with coordination numbers from 1 to 7. Adapted with permission from 37, Copyright 2012 Wiley. (C) Field emission scanning electron microscopy (FE-SEM) images and 2D electric field distribution of fabricated multi-petal flowers and arrays. Adapted with permission from 38, Copyright 2014 Wiley. (D) Schematic diagram of typical coupling nanostructure hot-spot. Adapted with permission from 22, Copyright 2017 American Chemical Society.

Chen *et al.* explored the hot-spot phenomenon associated with colloidal nanoparticle monomers, dimers, and trimers. The enhancing effect of hot-spot in dimers is mainly localized in the interparticle gap region, which accounts for the majority of the SERS signal. The SERS intensity of the trimer is 16 times higher than that of the monomer. Owing to its superior structural symmetry, the distribution of hot-spot in the trimer is more complex and abundant, resulting in enhanced signal amplification. The SERS intensity is 87 times higher than that of the monomer (**Figure 4A**) 35, and the varying configurations of trimers (linear, triangular) exert distinct influences on the intensity and dispersion of hot-spot.

An increased number of hot-spot can conspicuously enhance the intensity of the SERS signal, facilitating the evolution of SERS substrates from zero-dimensional to three-dimensional (3D) structures. Constructing plasmonic AuNPs into 3D configurations featuring dense metal nanostructures and nanogaps will enhance the hot-spot 36. This study involved the assembly of AuNPs into gold nanoclusters with various coordination numbers by the application of block copolymer coatings, which precisely maintained the interparticle spacing. The generated clusters were classified into dimers, trimers, tetramers, as well as intricate combinations of trigonal, octahedrons, and pentagonal bipyramids, presenting coordination numbers (CN) ranging from 2 to 7. Model fitting and experimental imply that suggest that as the CN increases from 1 (monomer) to 7 (pentagonal bipyramid), the intensity of SERS rises by 4000 times owing to numerous electromagnetic field couplings (**Figure 4B**) 37.

Mansoo Choi *et al.* utilized spark discharge and ion-induced electrostatic focusing to fabricate a nanoparticle aggregate structure referred to as “multi-petal flower assemblies”, which is characterized by diverse petals (**Figure 4C**) 38. The structure discloses the regulation of the plasma hot-spot in accordance with the variations in the number of petals m (4, 6, and 8). The increase of the petal count from 4 to 8 enables the plasma hot-spot attain SERS enhancement and multi-resonance properties of 10^7^ throughout the entire visible light spectrum, facilitating applications in in numerous novel plasma devices. A highly efficient SERS substrate, encompassing 3D hot-spot is autonomously fabricated through the “self-generated nanogap” technology. This substrate generates 3D nanogaps in both horizontal and vertical orientations, conspicuously enhancing the intensity of local electromagnetic field enhancement and the amount of adsorbed molecules. This significantly enhances the SERS intensity 39. Tian's research group employed coupled nanostructures (such as the aggregates of AuNPs, oligomers, gold core-satellite nanostructures, the vertical self-assembly of gold nanorods on a support, nanobump or nanovoid arrays were prepared by depositing Ag on the preassembled SiO_2_ or polystyrene spheres, nano-heptamers, and nano-cone tetramers as depicted in **Figure 4D**) 22. These coupled nanostructures display extremely intense hot-spot domains, and their average SERS intensity is typically 2 to 4 orders of magnitude higher than that of individual nanostructures. Recently, the utilization of face-to-face fabricated gold nanocubes has led to the optimization of their spatial configuration and distance, giving rise to hot-spot across several detection regions and forming a “wide hot-spot area”, thereby significantly enhancing both SERS and fluorescence. The sensitivity and efficiency of signal detection have facilitated precise tracking and quantification of miRNA-21 in cancer cells 40.

These coupled nanostructures generate high-density local electromagnetic fields. In comparison with single-particle structures, the average SERS signal intensity of these nanostructures is generally enhanced by 2 to 4 orders of magnitude. To evaluate more systematically the influence of different aggregation configurations on signal enhancement, we summarize in Table 1 the typical geometric parameters, the number of hot-spot, and the corresponding variation trends of the EF for AuNPs monomers, dimers, trimers, and multimers. This further reveals the “structure-hot-spot-function” correlation between the structural evolution and SERS performance.

The increase in the number of hot-spot directly enhances the intensity of the local electromagnetic field, thereby significantly improving signal sensitivity 41. This approach facilitates the detection of ultra-low concentrations of target molecules, rendering it particularly efficacious for trace analysis and biosensing 42. The preparation of more hot-spot typically relies on straightforward physical, chemical, or self-assembly techniques, such as particle aggregation, assembly, or template-assisted assembly, which are not technically complex and are readily executable. This stage has witnessed an increase in the number of hot-spot, however, many of these might be ineffective and exhibit restricted local field enhancement effects, thereby resulting in low resource utilization 43,44. The lack of precise control over the location and intensity of hot-spot constrains the need for greater precision and wider applications 45. The formation of numerous hot-spot typically relies on the aggregation of nanoparticles, however, the aggregation state of these particles may vary over time (e.g., precipitation, separation), resulting in an unstable enhancing effect of the hot-spot 46. The augmentation of disorder within hot-spot might give rise to the adsorption of non-target molecules in these regions, thereby leading to an elevated background noise and a reduced signal-to-noise ratio 47.

### 2.2 Control the structure of hot-spot

In this stage of the development of hot-spot technology, researchers will shift from enhancing additional hot-spot to constructing adjustable hot-spot, thereby enhancing performance through precise regulation of position, density, and intensity. The design of gold nanostructures with distinctive morphologies leads to the generation of enhanced hot-spot in targeted regions. When exposed to light stimulation, gold nanocavities, gaps, and bridge structures are capable of generating surface resonant plasmon coupling, thereby leading to the formation of highly intense plasmon hot-spot domains 48. The emergence of hot-spot regions in nanostructures is capable of amplifying the optical signal strength by 10^5^~10^8^ times, typically demanding nanocavities, gaps, and bridges within the range of 2 to 10 nm. Managing and synthesizing gold nanostructures with precise morphologies within the 10 nm scale to meet specific performance criteria constitutes a prevailing bottleneck in the contemporary fields of nano-optics, materials science, and optics.

**Figure 5:**
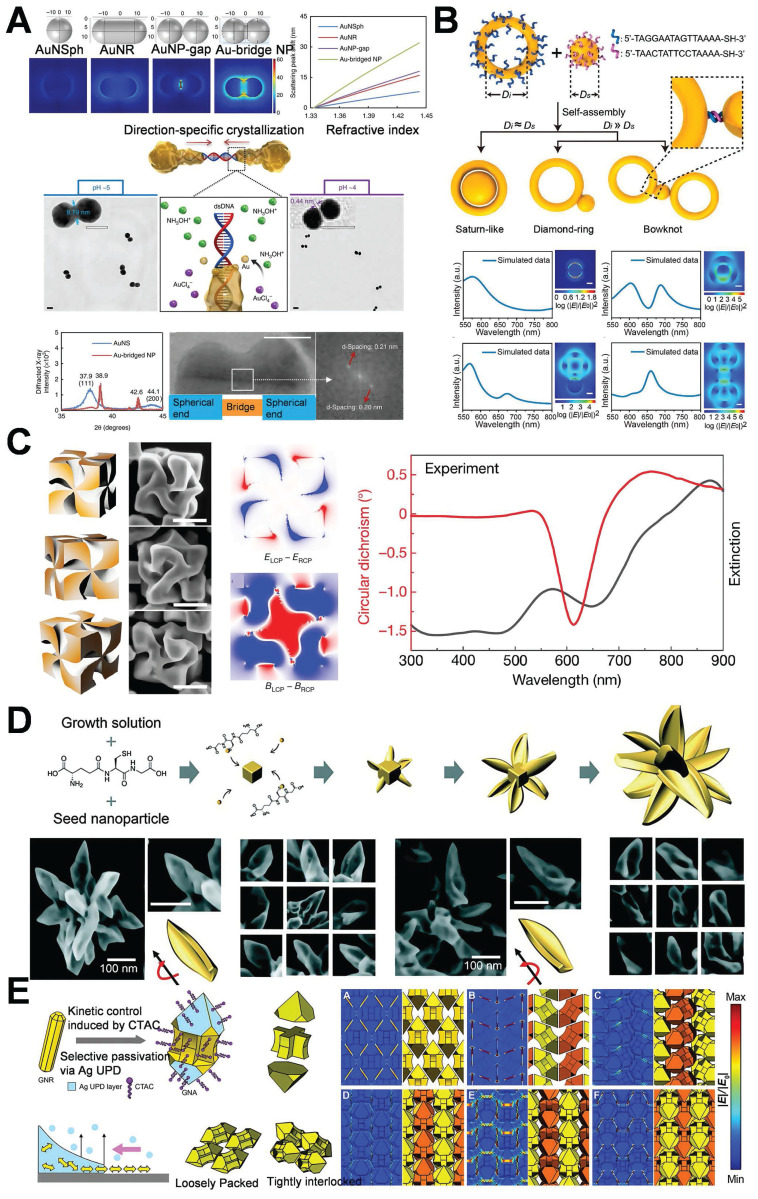
Schematic diagram of controlling the structure of hot-spot: (A) DNA-directed synthesis of gold nanobridges. Adapted with permission from 52, Copyright 2019 Springer Nature. (B) Schematic illustration of the shape-complementary regulated assembly of gold nanorings and nanospheres. Adapted with permission from 53, Copyright 2020 American Chemical Society. (C) Morphology and optical properties of chiral plasmonic AuNPs synthesized using amino acids and peptides. Adapted with permission from 59, Copyright 2020 Multidisciplinary Digital Publishing Institute. (D) Schematic diagram of the synthesis and morphology of an outward-facing petal-like structure with a two-dimensional curved surface. Adapted with permission from 60, Copyright 2023 Wiley. (E) Schematic illustration of the synthesis and self-assembly of gold nanoarrows and the distribution of the electric field. Adapted with permission from 64, Copyright 2017 American Association for the Advancement of Science.

The DNA-directed metallization of nanomaterials has emerged as a feasible approach to tackle these difficulties 49,50. Employing DNA as a metallization template enables a considerable degree of control over the characteristics of the synthesized nanomaterials 51. Ma *et al.* integrated the rigidity and charge characteristics of DNA with a precise control mechanism employing AuNPs, thereby promoting the technology for nonlinear directional synthesis of nanomaterials. This approach allows for the regulation of 2 nm bridges and 1 nm gap structures, significantly intensifying the electromagnetic field within the gap to generate robust hot-spot, effectively utilizes biological materials to tackle the challenges presented by chemical materials (**Figure 5A**) 52. Subsequent research presented a novel technique that employed form matching to control DNA-directed self-assembly for the fabrication of heterostructures with the utilization of gold nanorings. This study demonstrated that gold nanorings and nanospheres self-assemble into complex structures, such as “saturn”, “ring”, and “bowtie”, under the influence of form complementarity (**Figure 5B**) 53. DNA facilitates assembly through establishing complementary base pair interactions, allowing for the precise alignment of components. The emergence of hot-spot results from the intentional disruption of symmetry in these nanostructures. This symmetry breakdown gives rise to localized plasmon resonances and enables higher-order plasmonic modes. The gaps significantly enhance the electromagnetic field around the hot-spot, rendering these nanostructures beneficial for sensing and photonics applications. Researchers have successfully fabricated DNA-directed nanostructures in various geometries for enhancing hot-spot, such as nanoflowers, nanopolygons 54, dendritic formations 55, and sea cucumber-like configurations 56. Finite element modeling was carried out to quantitatively analyze the electromagnetic enhancement properties of these geometries under consistent excitation conditions (λ=633 nm, interparticle gap=2 nm). As depicted in** Figure 5B**, spherical dimers demonstrated the most robust dipole-dipole coupling. The maximum electric field enhancement |E/E₀|² reached 4.11×10⁹. Conversely, cubic dimers generated relatively weaker localized fields (|E/E₀|²≈1.24×10⁸), which were concentrated around their sharper edges owing to the lower curvature. Significantly, the asymmetric “bowtie” structure yielded a spatially extended distribution of hot-spot. The coverage of these hot-spot was approximately three times larger than that of their symmetric counterparts, emphasizing the impact of symmetry breaking on expanding field localization. These findings confirm that sharper features (such as cube edges) are positively correlated with field amplification, and structural asymmetry facilitates broader hot-spot activation regions.

In addition to DNA, amino acids and peptides are extensively employed in the guided synthesis of gold nanostructures, especially in enhancing hot-spot. An escalating number of researchers are utilizing amino acids and peptides to modulate the optical activity and chirality of AuNPs 57,58. In this study, peptides were utilized to facilitate the anisotropic growth of AuNPs, leading to the formation of twisted rods or helical structures (**Figure 5C**) 59 and thereby concentrating the electromagnetic field in a designated area. Peptides are capable of precisely regulating the configuration and distance of nanoparticles, thereby enhancing the intensity of hot-spot for applications such as chiral sensing. Nam *et al.* explored the function of peptide sequences in governing the assembly and growth of AuNPs. The study revealed that glutathione (GSH) promotes the formation of gold nanostructures featuring twisted petal-like structures during particle growth (**Figure 5D**) 60. Morphological control is of paramount importance for the design of substrates featuring specific hot-spot properties.

Furthermore, the hot-spot can be modulated by external stimuli, and their intensity and distribution can be modified through variations in the external environment. Lasers or alternative light sources are capable of generating heat effects or photoinduced deformations of AuNPs, thereby modifying the interparticle gaps 61. Alternatively, magnetic nanoparticles could be integrated with AuNPs, and magnetic fields are capable of manipulating the spatial arrangement of the nanoparticles to facilitate dynamic alterations in the hot-spot 62. External electric fields are capable of modifying the distribution or orientation of charged AuNPs, thereby dynamically adjusting the hot-spot regions 63. Qi *et al.* accomplished the precise control over the anisotropic growth of AuNPs to form gold nanoarrow structures via the seed-mediated growth approach in combination with morphology regulators. All the tip, bifurcation, or groove area of the gold nanoarrows can act as hot-spot. Subsequently, by modifying the self-assembly environment (such as pH and ionic strength), the spacing or orientation of the nanoarrows can be dynamically adjusted, thereby attaining controllable self-assembly. Through the trinity strategy of shape anisotropy, controllable self-assembly, and precise growth, this study achieved the flexible regulation of the spatial position, quantity, and intensity of the hot-spot (**Figure 5E**) 64.

**Figure 6:**
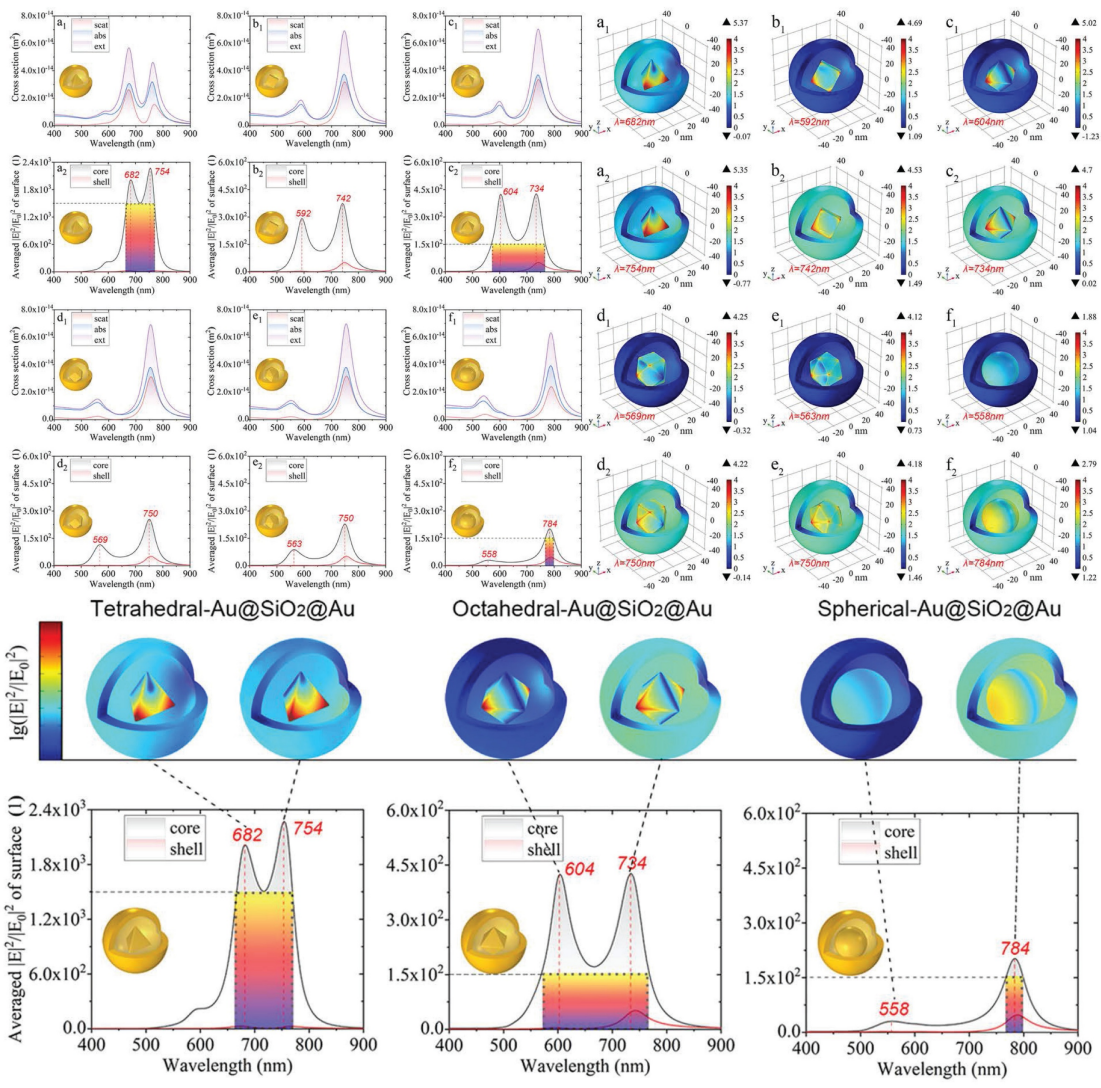
Schematic diagram of polyhedron-Au@SiO_2_@AuNPs finely controlling local hot-spot through modifying the morphology of the nanogap between the shell and the core. Adapted with permission from 67, Copyright 2022 American Chemical Society.

Simultaneously, it is feasible to construct multi-level or complex structural complexes with adjustable hot-spot, enabling the control over the distribution of these hot-spot through alterations to the overall structural characteristics. Develop core-shell architectures of AuNPs and other metals or dielectric materials, while modulating the shell thickness to precisely regulate the intensity of hot-spot 65,66. Qin *et al.* demonstrated the tunable local enhancement properties of hot-spot via polyhedral Au@SiO₂@AuNPs. Modifying the morphology of the nanogap between the shell and the core enables precise control of the local field enhancement (**Figure 6**) 67, providing versatility for a variety of scientific and technological applications.

The second stage of the hot-spot technology is intended to control the structure of hot-spot. The intensity and location of the hot-spot can be engineered and adjusted in accordance with requirements, thereby enhancing their flexibility and controllability, and facilitating the optimization of designs for specific applications. The development of adjustable hot-spot enables AuNPs to better satisfy the requirements of various application scenarios 68-70. The precise regulation of the position and intensity of the hot-spot continues to pose a technical challenge. Variability can still arise at the nanoscale, even when the experimental conditions are uniform. Ensuring the compliance of each hot-spot with the design requirements in large-scale preparations remains a challenging task. The performance of adjustable hot-spot is often affected by external factors such as ambient temperature, solvent, pH, as well as electric or magnetic fields. Consequently, precise control of experimental conditions is indispensable in practical applications, thereby augmenting operational complexity 71. While the hot-spot might be subject to regulation, such regulation tends to be static and presents challenges for real-time adjustments after preparation. This limitation restricts its *in vivo* application. Future research is centered on the development of more efficient dynamic regulation technology to rapidly and precisely adjust the hot-spot characteristics while guaranteeing system stability 72.

### 2.3 Efficient control of hot-spot utilizations

Although it is feasible to generate hot-spot featuring exceptionally high electric field strengths by means of advanced assembly or design, the signal enhancement is still restricted if the target analytes are not efficiently delivered to these areas. Furthermore, surface ligands often localize in hot-spot to guarantee stability and functionalization. These ligands might compete with analytes for adsorption at these sites or generate intense signals that obscure the signals of the target molecules. Consequently, the efficient delivery of target analytes to the hot-spot is of equal significance to constructing high-performance hot-spot. Both aspects need to be optimized synergistically in order to achieve optimal signal enhancement 73.

In recent years, DNA origami technology has attracted considerable attention 74,75. The programmability of the system facilitates precise control of metal nanostructures, enabling the accurate positioning of binding sites for target molecules on the DNA template. This design enables the delivery of target analytes to specific hot-spot areas, thereby leading to a significant enhancement of the signal and a notable improvement in the detection sensitivity of the target analyte, including the ability for single-molecule detection.

**Figure 7:**
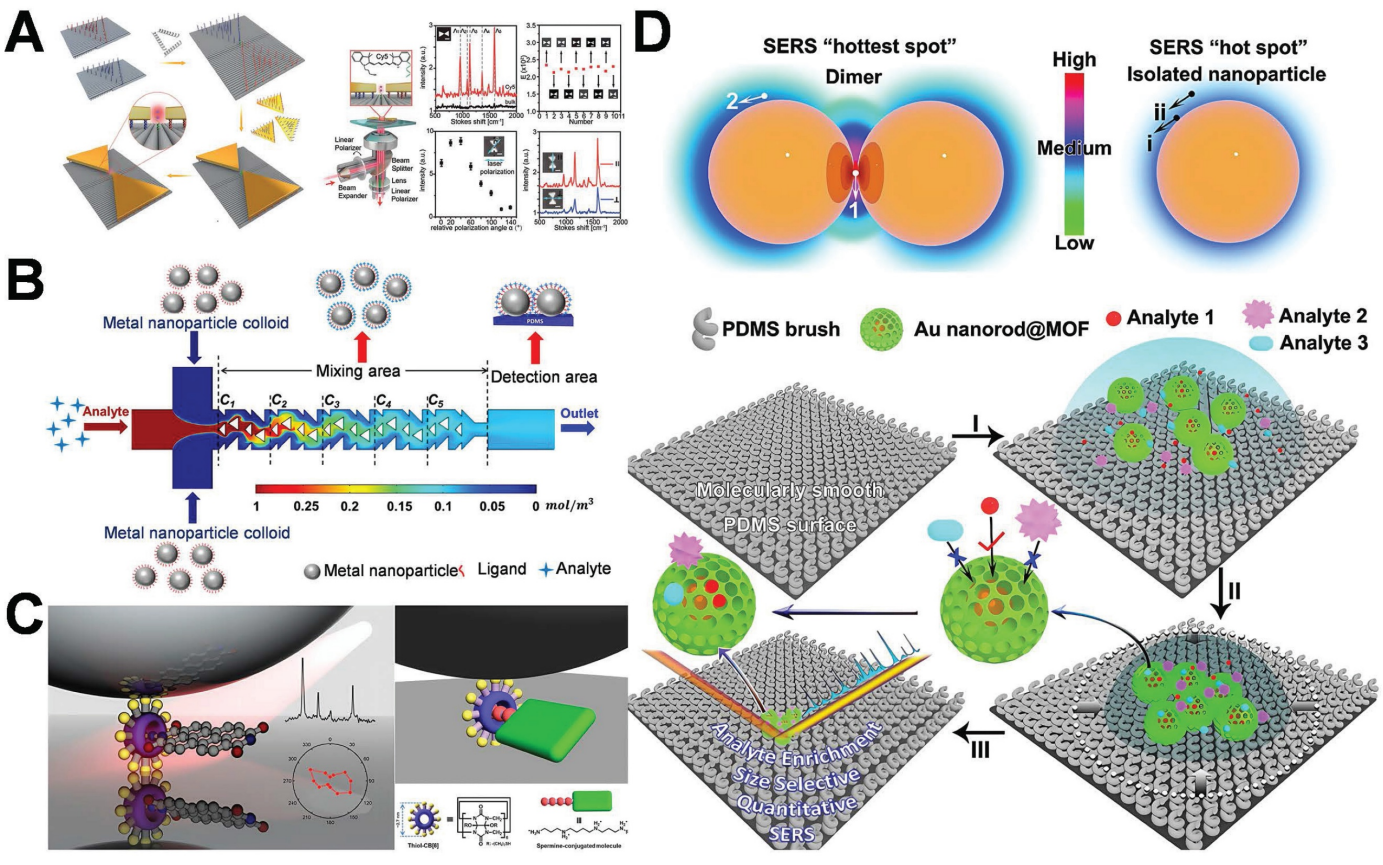
Schematic diagram of efficient control of hot-spot utilizations: (A) DNA origami templates for the precise construction of gold bow-knot nanoantennas and single-molecule SERS detection. Adapted with permission from 74, Copyright 2018 Wiley. (B) SERS technology based on a hybrid-assisted hot-spot occupation strategy microfluidic chip. Adapted with permission from 80, Copyright 2018 American Chemical Society. (C) Host-Guest Chemistry accurately locates single molecules at plasmon nanojunctions and effectively utilization of hot-spot. Adapted with permission from 81, Copyright 2018 American Chemical Society. (D) A comprehensive SERS platform integrating enrichment, filtration, and hot-spot enhancement. Adapted with permission from 82, Copyright 2020 American Chemical Society.

Ding *et al.* utilized DNA origami templates for the precise fabrication of a gold bowtie nanoantenna. The gold bowtie nanoantenna is composed of two gold nanotriangles. The nanoscale precision of DNA was employed to regulate the distance between two AuNPs within a range of 1-2 nm, thereby creating hot-spot areas with enhanced gap signal (**Figure 7A**) 74. Binding sites are precisely engineered on the DNA origami template for the capture and immobilization of target molecules. The target molecules are located within the gap regions of the gold bowtie, thereby exposing them to intense electromagnetic fields. This significantly enhanced the signal and facilitates detection at the single-molecule level. At this stage, microfluidic devices are utilized to precisely convey analytes to the hot-spot region through regulating fluid flow, while simultaneously minimizing the interference of non-target substances, thereby optimizing the utilization of the hot-spot 76-79. This research integrated hot-spot with microfluidic technology, generating hot-spot through the construction and design of AuNPs to ensure enhanced sensitivity. Microfluidic channels incorporate mixing components, like serrated or spiral structures, to enhance the contact probability between the analyte and the hot-spot. Hot-spot occupancy is achieved through a mixing-assisted approach and fluid dynamics regulation to boost detection sensitivity and efficiency (**Figure 7B**) 80. The physical design, entailing the contraction or expansion of the microfluidic channel, guides the analyte towards the hot-spot region, thereby minimizing the diffusion distance between the hot-spot and the analyte, which boosts the efficiency of the analyte's arrival at the hot-spot.

Numerous researchers utilize chemical adsorption techniques to functionalize and engineer specific binding molecules (such as biological receptors, antibodies, or molecular probes) in the hot-spot region, thereby preferentially concentrating the target analyte in the hot-spot through molecular recognition, facilitating the efficient utilization of the hot-spot. Kimoon Kim *et al.* utilized host-guest chemistry to precisely position single molecules at plasmonic nanojunctions, thereby efficiently harnessing the hot-spot and addressing the fundamental issue of hot-spot consumption (**Figure 7C**) 81. The host pre-modifies the metallic surface in order to establish a chemical “capture point” within the nanogap. The guest is guided into the hot-spot region and stabilized through host-guest interactions, circumventing the random molecular distribution that is inherently present in conventional chemical adsorption techniques. The work presented a distinctive architecture of a comprehensive SERS platform integrating enrichment, filtering, and hot-spot enhancement (**Figure 7D**) 82. The researchers optimized the nanostructure to enhance the uniformity of the distribution of hot-spot and improve efficiency; precisely enriched target molecules through electrostatic interaction or molecular recognition; and eliminated interfering molecules by designing nanopores and surface modification layers, effectively addressing the bottleneck issue of the conventional utilization of hot-spot.

This stage of hot-spot technology optimizes the effect of signal enhancement. The effective application of hot-spot technology boosts the detection sensitivity to the molecular level, facilitating single-molecule detection and analysis 82. Nevertheless, both the preparation and large-scale production confront technical obstacles. The current approaches are costly and demonstrate low reproducibility, thereby restricting their industrial application 83,84. The effective exploitation of the third phase of AuNPs hot-spot technology poses challenges and opportunities. The development of intelligent hot-spot is anticipated to become prominent in the future. The design utilizes computational simulation and machine learning techniques to predict and design nanostructures featuring an optimal distribution of hot-spot, providing a theoretical basis for the development of more efficient structures 85. Design a dynamically responsive hot-spot structure to facilitate the switching, distribution adjustment, and intensity optimization of hot-spot via the dynamic regulation of external stimuli such as light, heat, electricity, and magnetism, thereby accommodating various scenarios and enhancing the broader utilization of hot-spot technology in optoelectronic devices, biomedicine, environmental testing, and numerous other domains 86-88.

Despite the fact that advanced strategies have successfully realized hot-spot structures featuring ultra-high electromagnetic field enhancement capabilities, their practical utility in biomedical applications remains highly contingent upon their structural stability within physiological environments. Three primary challenges must be addressed. (1) Ionic strength-induced aggregation: in biological fluids (approximately 150 mM NaCl), electrostatic shielding may give rise to uncontrolled nanoparticle aggregation. Colloidal AuNP dimers or trimers lose their SERS activity in serum in the absence of surface modification 35. Moreover, multi-petal plasmonic structures display rapid resonance degradation under isotonic conditions 38. (2) Protein corona effects: upon entering the body, AuNPs tend to undergo non-specific adsorption with plasma proteins, thereby forming a protein corona with a thickness of approximately 5-10 nm. This corona layer not only impedes the entry of target molecules into the hot-spot enhancement region but may also induce structural rearrangement. The gold bowtie antenna fabricated through DNA origami exhibits a red shift of the plasmon peak in serum, suggesting a wider gap and a weakened hot-spot field 71. (3) Biodegradation vulnerabilities: several connection elements upon which hot-spot structures depend, such as DNA and aptamers, exhibit instability under physiological conditions. They are highly susceptible to degradation by nucleases, which ultimately results in the disintegration of these structures. The DNA linker arms in the nucleus-satellite structure can be degraded by enzymes present in the blood within a few hours 22.

In addressing these challenges, researchers have put forward a diverse range of structural stability optimization strategies. For instance, PEGylation modification can remarkably mitigate non-specific protein adsorption and extend the *in vivo* circulation time. It has been verified that it can sustain the SERS activity of hot-spot structures in serum for over 24 hours 89. Through the construction of a silica shell layer, it is feasible to inhibit particle aggregation while maintaining a sub-5 nm gap and preserve the accessibility of analytes 67. Moreover, pH-responsive design can be employed to accomplish *in vivo* in-situ assembly of hot-spot structures. For example, certain gold nano-arrow structures self-assemble to form hot-spot solely in the slightly acidic tumor microenvironment, thereby achieving high environmental adaptability 64. The integration of these strategies offers solutions for the practical implementation of hot-spot structures within complex biological settings, thereby significantly augmenting their potential for clinical translation.

Based on the above-mentioned analysis, the geometric configurations and local electromagnetic field enhancement capabilities of diverse gold nanostructures play a crucial role in determining their application performance within the biomedical domain. To conduct a systematic evaluation of the comprehensive performance of various hot-spot structures with respect to EF, synthesis complexity, and stability in the physiological environment, Table 2 showcases a parametric comparison of the current representative structural types. This is intended to offer a quantitative foundation and selection reference for structural optimization and clinical translation.

Gold nanostructures featuring diverse configurations establish a structural foundation for the precise modulation of the hot-spot effect and create advantageous conditions for their applications in the realm of biological detection. With the ongoing refinement of structural design and stability strategies, the application scope of hot-spot empowered AuNPs in the identification of tumor markers is steadily expanding. The subsequent text will concentrate on their specific applications in breast cancer diagnosis.

## 3. Application of hot-spot empowered AuNPs in the diagnosis of breast cancer

The three developmental stages of hot-spot technology related to AuNPs, namely quantity enhancement, structural control, and efficient utilization, have not only promoted the progressive advancement of the technology but also facilitated its application in the biomedical field. The hot-spot enhancing effect of AuNPs conspicuously amplifies the signals of LSPR and SERS, thereby elevating the sensitivity of breast cancer-related biomarker detection and facilitating the precise identification of early and low-concentration markers 41. The enhanced utilization of AuNPs hot-spot technology will promote its innovative application in the diagnosis of breast cancer, facilitate the advancement of early screening and precision medicine, and thereby conferring substantial clinical benefits to breast cancer patients.

### 3.1 Optical diagnosis of breast cancer

LSPR and SERS represent two exemplary optical detection techniques grounded in the hot-spot effect of noble metal nanoparticles. These techniques find extensive applications in highly sensitive biomolecule detection and early disease screening. LSPR pertains to the phenomenon where, upon irradiation of metal nanoparticles with light of a specific wavelength, the free electrons on their surface engage in collective oscillation. This process enhances the absorption and scattering of light, resulting in an observable shift in the wavelength of the resonance peak. This effect exhibits extreme sensitivity to the refractive index of the surrounding medium and is thus frequently employed to detect the binding events of marker molecules 90,91. SERS, likewise, exploits the same plasmonic hot-spot to amplify Raman scattering signals by 10⁶-10¹⁰-fold via electromagnetic enhancement and chemical enhancement 92. Both LSPR and SERS can attain substantial signal enhancement by fabricating AuNPs hot-spot structures, thereby facilitating the ultrasensitive detection of trace biomarkers.

#### 3.1.1 Diagnosis of breast cancer utilizing LSPR technology

LSPR is a distinctive optical phenomenon generated by metal nanoparticles upon interaction with light. The binding of breast cancer markers (such as HER2 protein and CA15-3 antigen) to the surface of functionalized AuNPs modifies the local refractive index, resulting in a red shift or intensity variation of the LSPR peak position. The alteration in the optical signal can be precisely identified and exploited for the quantitative evaluation of marker concentration. The hot-spot enhancing effect of AuNPs augments the optical response of the interaction between marker molecules and the surface of AuNPs, thereby significantly enhancing the detection sensitivity of breast cancer-related indicators 93.

**Figure 8:**
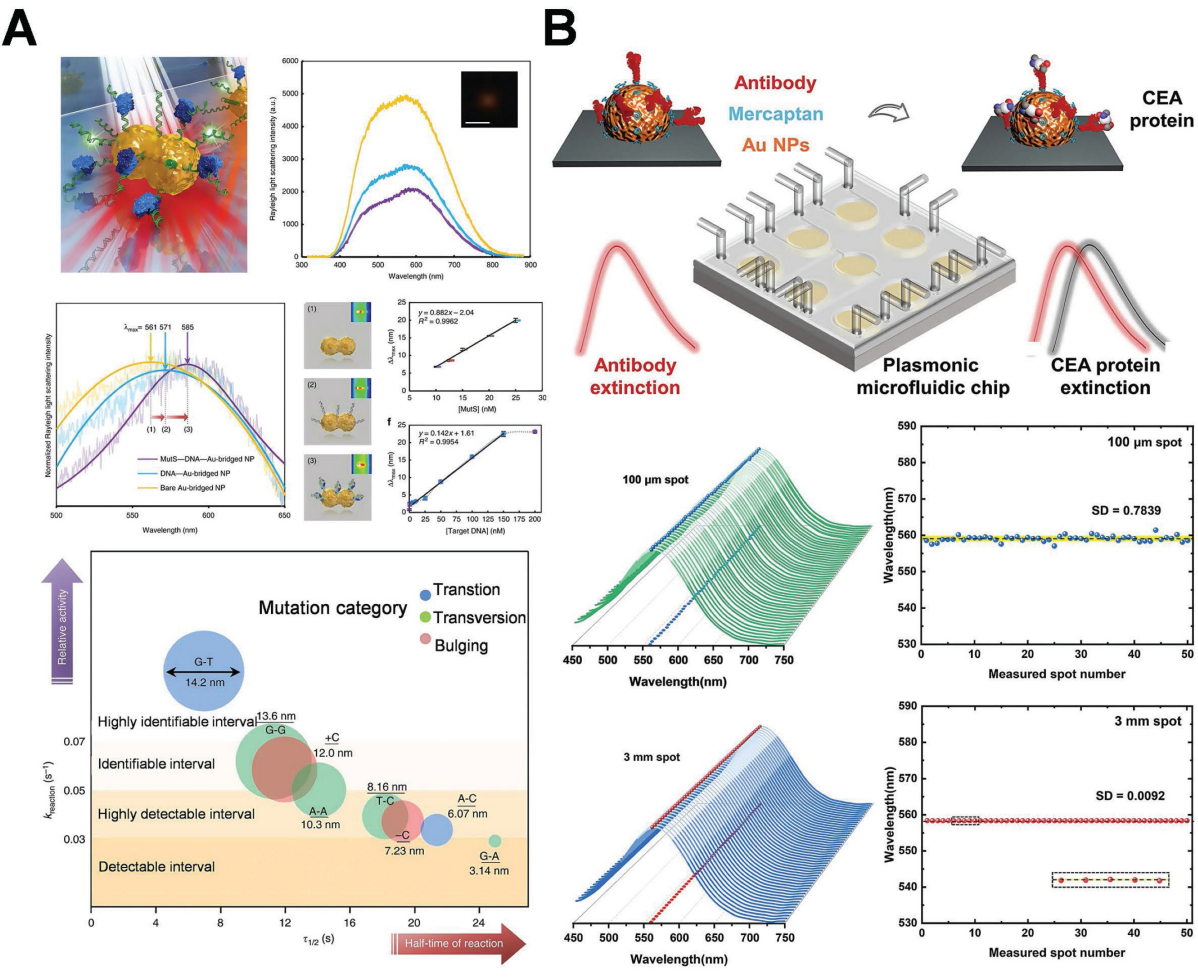
Schematic diagram of AuNPs combination of AuNPs and LSPR technology based on hot-spot enhancement for breast cancer diagnosis: (A) Single gold bridge nanoprobe for detecting breast cancer-related gene mutations. Adapted with permission from 52, Copyright 2019 Springer Nature. (B) Integrated microfluidic plasma chip based on AuNPs, employing LSPR technology to detect CEA in human serum. Adapted with permission from 94, Copyright 2022 American Chemical Society.

Ma *et al.* delineated a novel approach employing single gold-bridged nanoprobes, which enabled ultra-sensitive detection of single-point DNA mutations through the hot-spot amplification effect of LSPR signals (**Figure 8A**) 52. AuNPs integrated with LSPR technology have significant application in the diagnosis of breast cancer. This method enabled the sensitive detection of single-point DNA alterations through the hot-spot enhancing effect. It might identify subtle alterations in cancer-associated DNA sequences and target mutations in genes such as BRCA1/BRCA2, thereby facilitating early and precise detection in liquid biopsy samples, providing robust support for early breast cancer screening and corrective diagnosis. Researchers further integrate LSPR technology with microfluidic chips. LSPR technology is utilized to identify carcinoembryonic antigen (CEA) in human serum. Low concentrations of CEA (<1 ng/mL) can be identified, functioning as a crucial diagnostic indicator for the early stages of breast cancer (**Figure 8B**) 94. The microfluidic platform integrated with the LSPR sensor enables rapid, low-volume sampling and high-throughput detection. The chip facilitates real-time dynamic monitoring of the early indicators of breast cancer and is expected to be employed in clinical settings. Preliminary clinical investigations have provided additional evidence in support of this potential. For instance, Liu *et al.* reported that an LSPR-based microfluidic chip demonstrated 93% sensitivity and 97% specificity in the detection of CEA in 80 serum samples. This performance surpassed that of conventional ELISA, which had a sensitivity of 85% 94. Likewise, Lao *et al.* utilized a fiber-optic LSPR system for the detection of thrombin in whole blood. The system achieved a detection limit of 1 nM and showed 90% concordance with the results of qPCR 95.

Hot-spot empowerd AuNPs amplify the adjacent electromagnetic field, significantly enhancing the LSPR signal. Even breast cancer biomarkers of low concentration can be detected with high sensitivity, attaining detection limits of femtomolar (fM) or lower, thereby enhancing the potential for early diagnosis of breast cancer 96. The surface of AuNPs can be facilely modified with antibodies, aptamers, or nucleic acid probes to facilitate selective binding to molecules associated with breast cancer. Multi-target detection can be achieved by establishing multi-marker arrays to improve diagnostic accuracy. This technique does not require additional markers (such as fluorescent or radioactive probes). The detection can be achieved by directly observing alterations in LSPR signals. It is capable of monitoring the real-time variations of tumor markers and providing immediate diagnostic feedback 93,97. Nevertheless, there exist specific impediments related to the application of LSPR technology based on hot-spot enhancement for the detection of breast cancer. The hot-spot phenomenon of AuNPs is contingent upon the meticulous design of particle spacing and nanostructure. Even minor geometric flaws or environmental modifications can significantly influence the LSPR signal, thereby reducing the sensitivity and reliability of detection 98,99. Environmental factors might exert an influence on the modified molecules on the surface of AuNPs, leading to particle aggregation or functional loss, which has an impact on detection stability. Moreover, ensuring consistent manufacturing of successive batches of AuNPs is a challenging task 100. Future technological progressions, encompassing enhanced nanostructure design and sophisticated analysis, are indispensable for the development of a more precise, efficient, and user-friendly breast cancer detection platform.

#### 3.1.2 Diagnosis of breast cancer utilizing SERS technology

In recent years, SERS technology has emerged as a vital tool for biomedical diagnostics due to its remarkable sensitivity and specificity in molecular fingerprinting. In the detection of breast cancer, hot-spot empowered AuNPs have manifested considerable potential as an effective SERS substrate. Exosomes hold considerable potential as novel biomarkers in liquid biopsies, however, accurately diagnosing various molecular subtypes of breast cancer remains a significant issue due to the challenge of discriminating their subtle compositional differences within complex clinical settings. To address the challenge of precisely identifying distinct molecular subtypes of breast cancer within a complex clinical context, the SERS technology, capitalizing on its highly sensitive detection capabilities for trace molecules and the specific recognition advantage of the “molecular fingerprint,” has increasingly demonstrated unique potential. By leveraging the LSPR effect, the SERS substrate fabricated on AuNPs can significantly enhance the Raman scattering signals of molecules within the hot-spot regions. The EF can reach an impressive magnitude of 10⁶-10¹⁰ times, enabling the accurate capture and identification of even minute amounts of breast cancer-related biomarkers 101,102. In typical breast cancer analysis samples, such as serum exosomes, cell lysates, and miRNA probe complexes, various molecular species all exhibit highly reproducible characteristic peak positions. For example, distinct peaks of the HER2 protein can be detected at 1002 cm⁻¹ (symmetric stretching of the benzene ring C-C bond), 1445 cm⁻¹ (bending of the lipid CH₂ group), and 1655 cm⁻¹ (amide I band) 103,104. Exosomal membrane proteins, including CD63 and epithelial cell adhesion molecule (EpCAM), display characteristic signals at positions such as 732, 1125, and 1580 cm⁻¹ 105,106. Upon the binding of the miRNA-21 probe, enhancements are frequently observed in the 721 and 1336 cm⁻¹ bands 107. By capitalizing on the intensity and combination of these “fingerprint-like” spectral features, researchers can effectively distinguish among different subtypes of breast cancer, thus improving the accuracy of liquid biopsy. Furthermore, to enhance the interpretability of spectra and their clinical applicability, statistical and artificial intelligence algorithms have been increasingly integrated for auxiliary analysis in recent years 108.

**Figure 9:**
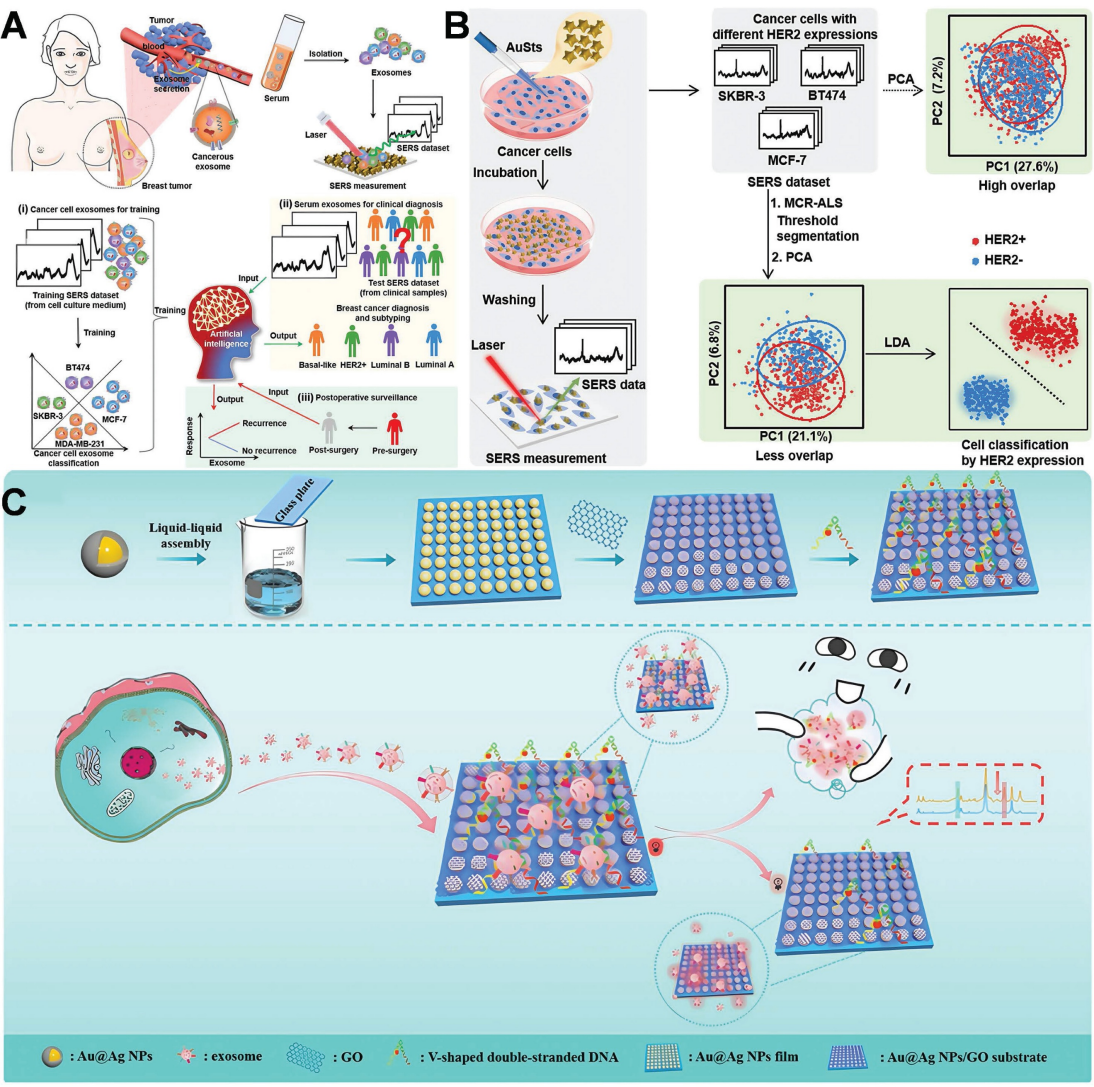
Schematic diagram of the combination of AuNPs and SERS technology based on hot-spot enhancement for breast cancer diagnosis: (A) AI label-free serum exosome SERS analysis for breast cancer diagnosis and postoperative evaluation. Adapted with permission from 109, Copyright 2022 American Chemical Society. (B) Label-free plasmon-enhanced HER2 spectroscopy for monitoring dynamic treatment of breast cancer. Adapted with permission from 112, Copyright 2022 American Chemical Society. (C) Ratio SERS biosensor for the detection of exosomes. Adapted with permission from 113, Copyright 2023 American Chemical Society.

Yu *et al.* proposed an artificial intelligence (AI)-SERS methodology that precisely predicted the SERS spectra of serum exosomes from clinical samples of various breast cancer subtypes, attaining 100% accuracy without surgery by assessing the label-free SERS spectral data of exosomes (**Figure 9A**) 109. The AI-SERS strategy utilized in this study is capable of discriminating serum exosomes from diverse pathological sources by detecting minute variations in spectral characteristics, while simultaneously addressing the challenges of complex spectral patterns, insufficient repeatability, and signal fluctuations typically witnessed in SERS measurements of clinical samples, thereby demonstrating remarkable clinical applicability. Building on this foundation, a study introduced a federated learning-enhanced SERS platform. It reached attomolar sensitivity for cancer exosome detection and protected patient data privacy among institutions. This federated AI-SERS framework has translational potential for breast cancer liquid biopsy, allowing ultra-sensitive, cross-center subtype classification via multi-site spectral aggregation 110. Although AI-assisted SERS profiling can yield population-level biomarker signatures, single-molecule detection platforms are filling the resolution gap. A recent study demonstrated tracking HER2 monomers at 0.1-1 pM using DNA-origami-tuned plasmonic nanocavities, enabling real-time monitoring of trastuzumab resistance via single receptor conformational dynamics 111.

In parallel, the researcher developed a machine learning-based SERS integration technique that targeted the inherent SERS properties of cancer cells to perform label-free detection of HER2 expression at the cellular level (**Figure 9B**) 112. The engineered plasmonic gold nanostars manifested superior SERS enhancement capabilities and were internalized by cancer cells through receptor-mediated endocytosis, augmenting the intrinsic SERS signal of the cells. They successfully identified and classified three distinct breast cancer cell lines with varying HER2 expressions with an accuracy of up to 99.6%.Another investigation utilized a dual-aptamer-assisted ratiometric SERS sensor for the ultrasensitive and precise detection of breast cancer exosomes (**Figure 9C**) 113. AuNPs were engineered to serve as hot-spot for Raman signals. After exosome capture, extensive cavities emerged on the surface of AuNPs. These regions are indispensable for the amplification of SERS signals. By employing two distinct aptamers targeting the characteristic proteins of exosomes (CD63, EpCAM), the synergistic effect of the dual aptamers enabled the precise capture of breast cancer exosomes and minimized the interference from nonspecific binding. The detection range spans from 2.7×10² to 2.7×10⁸ particles/mL, with a detection limit of 1.5×10² particles/mL, facilitating outstanding differentiation of exosome samples between breast cancer patients and healthy individuals. Achieved ultrasensitive and precise detection of breast cancer exosomes, providing a distinctive approach for the early diagnosis of breast cancer. A recent study employed SERS technology on 3D cell models, facilitating the detection of molecular characteristics both within and outside cells with remarkable sensitivity and resolution. This advancement enables an in-depth exploration of the onset, progression, and alterations in the microenvironment of breast cancer, thereby opening up a novel path for cancer research. SERS technology facilitates real-time microenvironment monitoring within 3D tumor models, highlighting its potential application in exploring dynamic cellular processes 114.

In contrast to conventional detection techniques (such as tissue biopsy or immunohistochemistry), SERS enables rapid and non-destructive molecular diagnosis of samples, thereby significantly enhancing the detection efficiency. Clinical investigations have further authenticated the translational potential of SERS. Serum-based exosome profiling attained 100% classification accuracy for breast cancer subtypes (n=50), exceeding the sensitivity of conventional IHC (85-90%) 109. A dual-aptamer SERS sensor achieved a detection limit of 1.5×10² particles/mL in clinical serum, outperforming ELISA by an order of magnitude 113. Although promising, more extensive clinical implementation necessitates enhanced inter-laboratory reproducibility and large-scale validation.

The combination of AuNPs and SERS technology can be integrated with microscope imaging techniques for imaging cancer cells or tumor microenvironments, thereby facilitating the observation of cancer progression 98,102,115. The distribution and intensity of hot-spot have a direct impact on the reliability of SERS measurements. Accurately regulating the assembly of AuNPs to achieve consistent enhancement effects of hot-spot poses a considerable challenge in the implementation of this technology. Advancements in the design of nanostructures for hot-spot continue to be a primary focus for future research 68,116,117.

The identification of SERS is impeded due to the lack of standardized signal normalization and data analysis protocols, which complicates inter-laboratory comparisons and hinders clinical application. Consequently, it is of paramount importance to establish reliable SERS data analysis standards for the advancement of technical standardization. It is of crucial significance to conduct more clinical investigations to determine the feasibility and efficacy of the technology 102,118,119. Simultaneously, the progress of portable SERS detection devices for point-of-care diagnostics, in conjunction with the application of microfluidic chips and portable spectrometers to reduce costs and improve operational convenience, poses challenges that need to be tackled in future research, ultimately resulting in the establishment of a high-sensitivity, cost-efficient, and multifunctional platform for precise breast cancer diagnosis 41.

### 3.2 Combination of optical and other technology for the diagnosis of breast cancer

In the current research, diverse technologies, in conjunction with optical technology, are utilized for the accurate diagnosis of breast cancer. Electrochemical sensors enable real-time monitoring through quantifying current, potential, or resistance variations. The electrode surface, modified with AuNPs, is capable of concentrating target molecules, thereby enhancing the detection performance. AuNPs synergistically amplify weak signals through the hot-spot, enhancing the effect and electrochemical reactions and facilitating the reliable detection of ultra-low concentration breast cancer biomarkers. The researchers fabricated a surface plasmon-coupled electrochemiluminescence (SPC-ECL) sensor characterized by robust hot-spot. The AuNPs were asymmetrically functionalized with mPEG-SH and thiol-DNA to facilitate the assembly of two AuNPs into dimers. The gold nanodimer is capable of significantly amplifying the ECL signal. Consequently, the researchers devised a sensing system employing the SPC-ECL mode for the identification of BRCA1, featuring a detection range from 1 fM to 1 nM and a detection limit of 0.83 fM 120. This LSPR-enhanced SPC-ECL platform also shows promising clinical relevance. In a cohort of serum samples (n=50), it demonstrated 95% concordance with tissue biopsy for BRCA1 detection, with a 100-fold lower detection limit compared to FISH assays. Such integration highlights the translational potential of LSPR-based multimodal diagnostics in liquid biopsy.

**Figure 10:**
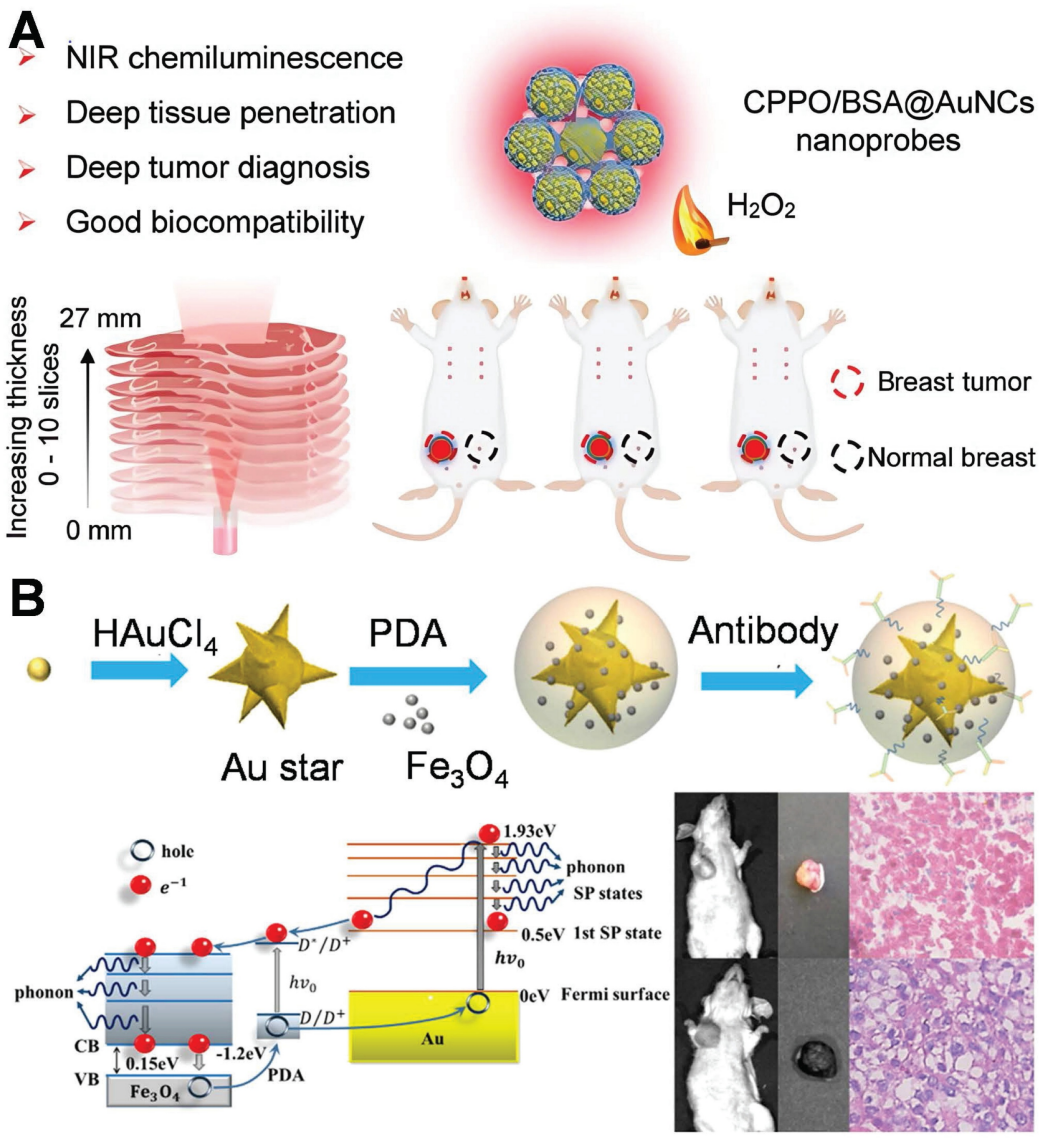
Schematic diagram of the combination of optical and other technology for the diagnosis of breast cancer: (A) Self-illuminating near-infrared chemiluminescence nanosensors for tumor imaging. Adapted with permission from 121, Copyright 2024 American Chemical Society. (B) Multifunctional nanoprobe based on gold nanostars@PDA@Fe₃O₄ for integrated tumor diagnosis. Adapted with permission from 122, Copyright 2021 Elsevier.

DNA-regulated gold dimers give rise to nanoscale gaps that generate highly localized electric fields, significantly enhancing the sensitivity of optical signals. Building upon this principle, researchers have developed multimodal diagnostic strategies that integrate optical techniques such as surface plasmon resonance (SPR) with electrochemistry and molecular biology, thereby enabling precise and ultrasensitive detection of breast cancer-related genes. Expanding on this approach, a self-illuminating near-infrared chemiluminescence (NIR-CL) nanosensor was fabricated (**Figure 10A**) 121. This nanosensor exploits chemiluminescence resonance energy transfer (CRET) to generate autonomous NIR emission without the need for external light excitation. This innovation effectively minimizes background autofluorescence and improves tissue penetration, facilitating high-contrast imaging of tumor sites. The integration of nanotechnology, chemiluminescence, and optical imaging in this system serves as an exemplification of the increasing potential of integrated diagnostic platforms for non-invasive, sensitive, and clinically translatable breast cancer detection.

Recently, an increasing number of studies have focused on multimodal diagnosis. AuNPs can be integrated with magnetic resonance imaging (MRI), Photoacoustic imaging (PAI), and SERS technologies to establish a multimodal diagnostic platform, facilitating comprehensive characterization from the micro to the macro levels and providing more extensive information for the diagnosis of breast cancer. This study investigated a multifunctional nanoprobe consisting of gold nanostars@PDA (polydopamine)@Fe₃O₄ for comprehensive tumor diagnosis (**Figure 10B**) 122. The hot-spot of gold nanostars stem from their dissimilar tips and angular configurations. The PDA coating layer augments the stability of the nanostructure and facilitates functional integration through modulating optical characteristics. Fe₃O₄ nanoparticles endow the system with MRI capabilities. The utilization of PDA@Fe₃O₄ facilitates multimodal diagnostic functionalities in MRI imaging and SERS detection, thereby enabling a more comprehensive characterization of the extent and localization of breast cancer lesions. This study achieved the diagnosis of breast cancer by means of the profound integration of optics (SERS/photothermal) with magnetism and nanomedicine. In analogous multimodal systems, SERS-assisted platforms have exhibited a diagnostic concordance of over 90% with conventional histopathology in breast cancer tissues. This finding underscores their potential clinical applicability, highlighting the significant promise these platforms hold in the realm of medical diagnostics.

## 4. Application of hot-spot empowered AuNPs in the therapy of breast cancer

The application of AuNPs in breast cancer therapy with hot-spot enhancement constitutes a pioneering progress in nanomedicine and PTT. This method exhibits superior efficacy in the diagnostics and treatment of breast cancer by capitalizing on the physical and chemical attributes of AuNPs and their specific interaction with the tumor microenvironment.

### 4.1 Targeted drug delivery

**Figure 11:**
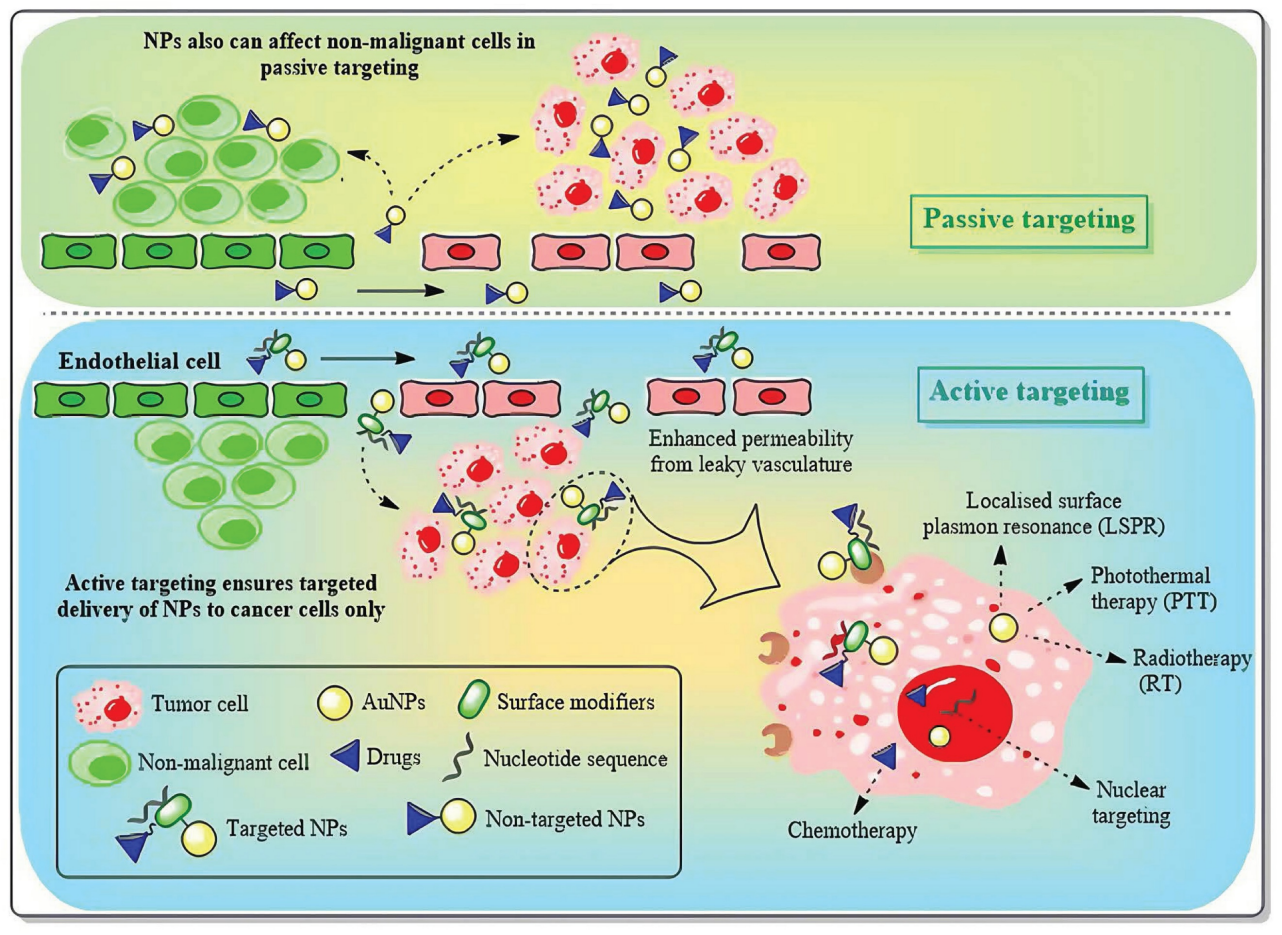
Active and passive targeting approaches with AuNPs in breast cancer treatment. Adapted with permission from 123, Copyright 2024 Royal Society of Chemistry.

As nanomedicine progresses, AuNPs assume an increasingly prominent role in tumor therapy. The targeting of AuNPs to cancer cells without causing damage to healthy cells can be accomplished through two distinct targeting approaches, namely passive targeting and active targeting (**Figure 11**) 123. Passive targeting primarily depends on the pathophysiological traits of tumor tissues. This delivery approach, facilitated by the high permeability of tumor blood vessels and the incomplete lymphatic drainage system (namely, the enhanced permeability and retention effect, EPR effect), allows AuNPs with a particle size ranging from 10 to 200 nm to selectively accumulate in tumor tissues. By modifying the surface with hydrophilic polymers like PEG, the blood circulation time of AuNPs can be notably prolonged, while the likelihood of being cleared by the reticuloendothelial system is decreased 124. Studies have indicated that AuNPs with a particle size between 50 and 100 nm exhibit the most ideal tumor accumulation effect. Nevertheless, this delivery method has considerable limitations, including individual variations in the EPR effect due to tumor heterogeneity and potential non-specific distribution. Even so, passive targeting, given its advantages such as straightforward preparation and good stability, remains one of the most extensively studied delivery strategies at present 125.

In contrast to passive targeting, active targeting delivery modifies specific targeting molecules (such as monoclonal antibodies, nucleic acid aptamers or small molecule ligands) on the surface of AuNPs, allowing them to actively recognize and bind to the specific receptors that are overexpressed on the surface of tumor cells 124. In the treatment of breast cancer, commonly utilized targeting ligands encompass trastuzumab targeting the HER2 receptor, folic acid molecules targeting the folate receptor, and RGD peptides targeting integrins, among others. This delivery approach can not only markedly enhance the accumulation efficiency of nanoparticles at the tumor site but also facilitate the endocytosis of tumor cells, thereby augmenting the therapeutic effect and reducing systemic toxicity 126. The most recent research also endeavors to combine active targeting with stimulus-responsive drug release systems, such as constructing pH-sensitive or enzyme-sensitive nanocarriers to achieve more precise drug controlled release 126,127. Nevertheless, this strategy still confronts challenges regarding the stability of targeting molecules and immunogenicity. With the advancement of nanotechnology and molecular biology, active targeting delivery is anticipated to become a crucial breakthrough in the precise treatment of breast cancer.

In the design of the AuNPs drug delivery system, geometric structure parameters, primarily size and shape, serve as the core variables that dictate its *in vivo* behavior and therapeutic efficacy. These two parameters respectively exert a critical influence on the biodistribution, cell uptake mechanism, circulation stability of nanoparticles, and the hot-spot effect. Thus, they necessitate collaborative optimization during the design phase. It is commonly accepted that nanoparticles within the 10-200 nm range can attain enrichment facilitated by the EPR effect in tumor tissues. Among these, 50-100 nm AuNPs are extensively regarded as the most appropriate for passive targeting drug delivery. These AuNPs can circumvent glomerular filtration (<10 nm) and, to a certain degree, elude clearance by the immune system (>200 nm are more readily recognized by the phagocytic system). Moreover, the smaller the particle size, the larger the surface area, which proportionally enhances the drug loading capacity and release rate. Conversely, larger-sized particles contribute to an extended circulation time and greater carrier stability 128. Consequently, a rational selection of size parameters serves as the foundation for modulating the system exposure time, pharmacokinetic behavior, and tissue penetration ability. On the premise of ensuring controllable dimensions, the morphological variations of AuNPs exert a direct influence on their local electromagnetic field distribution and biological interaction capabilities. Consequently, these morphological characteristics determine the diverse functions of AuNPs in aspects such as imaging enhancement, photosensitive response, and drug-loading modalities. Gold nanospheres, characterized by a uniform surface and a stable structure, serve as the most prevalently used basic drug-loading architectures. They are amenable to surface modification and static loading. Nevertheless, owing to their relatively low surface curvature and negligible hot-spot effect, the local electromagnetic enhancement capacity of gold nanospheres is constrained. Consequently, they are predominantly employed for stable drug delivery rather than for enhancing functionality 129. Gold nanorods with an aspect ratio of 3-5 exhibit a longitudinal plasmon resonance peak at 800-1000 nm. The local hot-spot formed at the termini of their longitudinal axes can enhance the release performance in response to laser irradiation. Moreover, they can support excitation within the near-infrared band, thereby enabling photothermal combined therapy. Additionally, the high aspect ratio structure of gold nanorods can prolong the blood half-life and enhance the cell membrane penetration capacity 130. Gold nanostar consists of multiple sharp protrusions. The tips represent regions where electromagnetic hot-spot are concentrated, rendering it an optimal structure for attaining high-intensity SERS signals and local thermal effects. Moreover, its irregular morphology can further augment cell membrane perturbation and endocytosis efficiency, thereby making it highly suitable for the construction of an integrated imaging-therapy platform 131. Gold nanocages possess a hollow and porous architecture. This architecture not only endows a high drug-loading capacity but also renders the edges of its shell liable to concentrate electromagnetic hot-spot. Moreover, it can be tailored to pH- or enzyme-responsive release mechanisms. This architecture enables selective degradation within the acidic microenvironment of tumors, thereby enhancing the precision of drug release 132. Gold nanoflowers and gold nanourchins exhibit intricate surfaces and multi-level protrusion architectures, thereby forming dense hot-spot distribution areas. Moreover, their highly rough surfaces not only enhance the adhesion to cell membranes but also boost the drug-loading capacity on the surface 133. In the construction of drug delivery systems, the size and morphology of AuNPs are not independent variables; instead, they are two pivotal elements for the coupled regulation of the functional structure. Through precisely modulating the size to optimize the *in vivo* kinetic behavior and integrating the morphology regulation of the hot-spot effect to enhance the functional response performance, it becomes feasible to offer a more robust design foundation and theoretical backing for intelligent drug delivery within the intricate tumor microenvironment 134-136.

Owing to its distinctive physicochemical properties and focused modification capabilities, AuNPs has manifested considerable advantages in the targeted delivery of breast cancer drugs. They not only enhance the efficiency but also the precision of drug delivery. Nevertheless, there exists a limitation in the studies within the literature regarding the application of AuNPs exploiting hot-spot enhancement for targeted drug delivery in single breast cancer. At present, the majority of research on AuNPs regarding hot-spot enhancement lays emphasis on their multifunctionality, which is elaborated in section 4.3 of this review. The limited research on single drug administration highlights the enhancing characteristics of hot-spot. The direct implementation of the hot-spot enhancing effect in the administration of a single drug for breast cancer remains challenging. The correlation between drug release and the enhancing effect of the hot-spot remains insufficiently elucidated. The hot-spot enhancing effect is strongly correlated with the particle size and shape of AuNPs, and optimizing these parameters is highly complex for the design of drug delivery systems. In the future, a comprehensive analysis of the hot-spot that enhance the impact, in combination with the attributes of the tumor microenvironment, is expected to lay the foundation for novel progress in drug delivery research 137,138.

### 4.2 Photothermal therapy and Photodynamic therapy

In recent years, phototherapy has increasingly emerged as the predominant approach for tumor treatment. Phototherapy mainly encompasses PTT and PDT. The essence of PTT lies in a photothermal agent (PTA). When exposed to external light sources such as near-infrared (NIR), PTA is capable of converting light energy into thermal energy, thereby eliminating tumor cells. AuNPs have emerged as the optimal PTA in PTT owing to their superior photothermal conversion efficiency, favorable biocompatibility, and adjustable optical properties. Hot-spot can significantly enhance the local electromagnetic field, thereby increasing the photothermal conversion efficiency of AuNPs, which is beneficial for the application of PTT 139. When AuNPs are exposed to light whose wavelength matches that of their surface plasmon absorption band, the surface electrons of AuNPs are excited and resonate vigorously, leading to the rapid conversion of light into heat. Cancer cells can be elevated to 41-47°C within minutes and killed by high temperatures during photothermal ablation 140,141.

**Figure 12:**
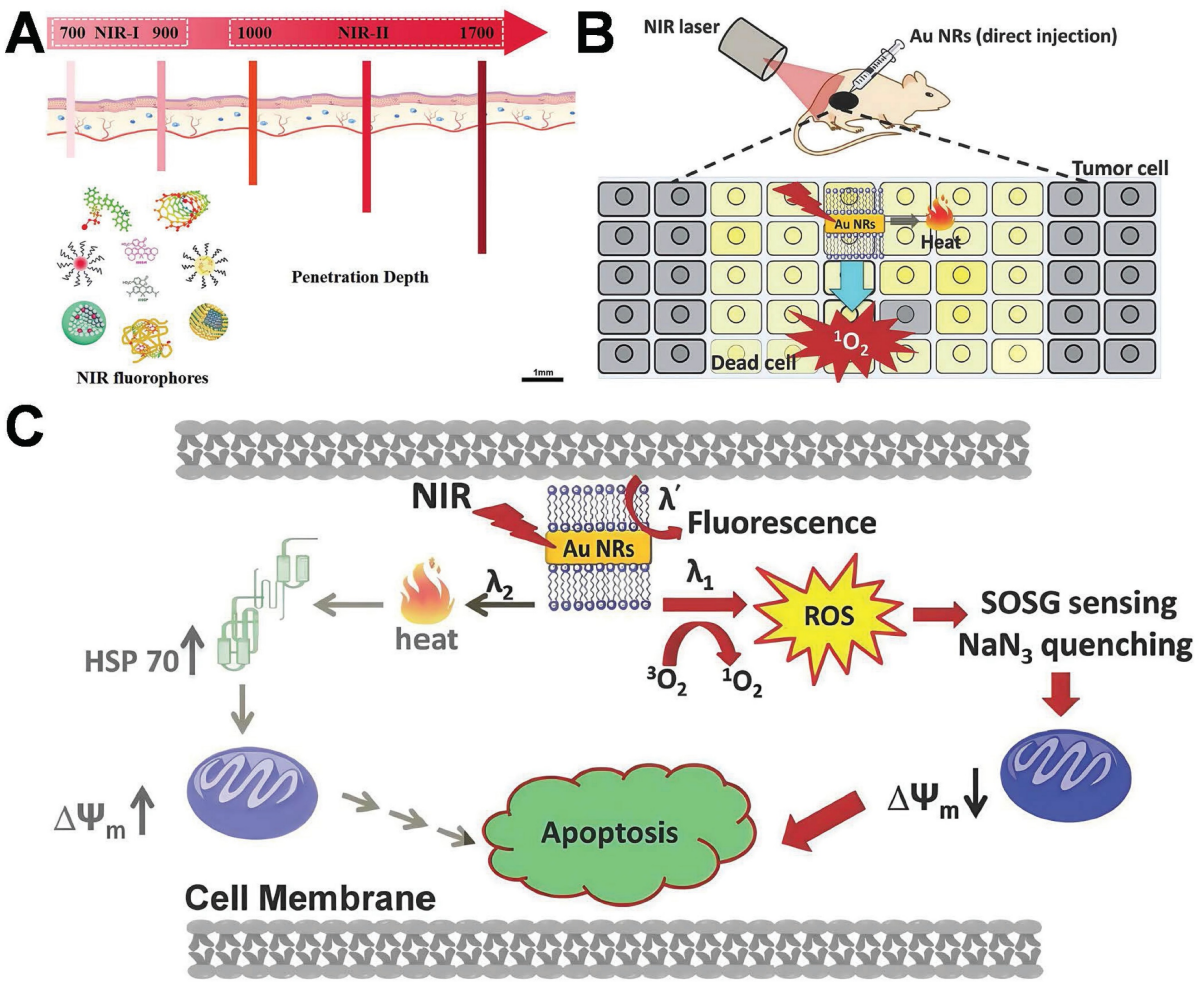
Schematic illustration of wavelength-dependent PTT/PDT mechanisms mediated by gold nanorods: (A) Penetration depth comparison between NIR-I (750-900 nm) and NIR-II (1000-1700 nm) in breast tissue. Adapted with permission from 142, Copyright 2021 Frontiers. (B) *In vivo* photo-destruction of malignant tumors. (C) Series of cellular events are involved in the PDT and PTT-induced cellular deaths upon photo-excitation of cells internalized gold nanorods. Adapted with permission from 144, Copyright 2014 Wiley.

The choice of excitation light wavelength stands as one of the pivotal factors dictating the efficacy of PTT and PDT. The NIR-I (750-900 nm), which are currently extensively employed in clinical practice, despite possessing high biological safety and imaging capabilities, typically exhibit a penetration depth of less than 3 mm in tissues. Conversely, the NIR-II (1000-1700 nm), owing to its reduced tissue scattering and lower water absorption, can achieve a penetration depth of 5-10 mm in biological tissues while concurrently maintaining excellent spatial focusing ability (as depicted in **Figure 12A**) 142. This physical characteristic offers a more effective optical treatment approach for deep-seated solid tumors, particularly malignancies such as breast cancer that are located deep within the body and are challenging to be fully covered by conventional treatment modalities. Consequently, in recent years, NIR-II responsive AuNPs have been the subject of extensive research. As an emerging platform for the precise PTT/PDT of deep tumors, they demonstrate promising application prospects.

Pakravan *et al.* demonstrated that numerous factors, including shape and light absorption efficacy, exert an influence on the heating efficiency of AuNPs 143. Nanoparticles with hot-spot architectures, possess superior photothermal conversion efficiency in comparison to other shapes. The principle of PDT lies in the generation of reactive oxygen compounds (ROS) by photosensitizers (PSs) upon exposure to specific light, which is capable of inducing cytotoxicity in tumor cells. PSs constitute a fundamental element in cancer therapy. When exposed to light of a specific wavelength, they are capable of generating reactive oxygen species (such as singlet oxygen, ^1^O_2_), which can induce apoptosis or necrosis in tumor cells. Under the circumstance of a low light dose, the working mechanism of gold nanorods via PDT/PTT-mediated cell death is depicted in **Figure 12** 144. Under light irradiation, gold nanorods can generate heat or singlet oxygen (or both) *in situ*, contingent upon the excitation wavelength (λ1, λ2 or λ') (**Figure 12C**). ^1^O_2_ and ROS formed subsequently can inflict direct damage on biological species, depolarize the mitochondrial membrane potential, and ultimately effectively induce apoptosis of cancer cells. When another excitation light (λ2) is employed, gold nanorods will convert the majority of the photon energy into heat, leading to an increase in the temperature of the local tumor site, the production of heat shock protein 70 (HSP 70), and the gradual initiation of the apoptotic process. PDT pathway is more efficacious than the photothermal therapy pathway in triggering the apoptotic process and subsequent cell death 144,145.

**Figure 13:**
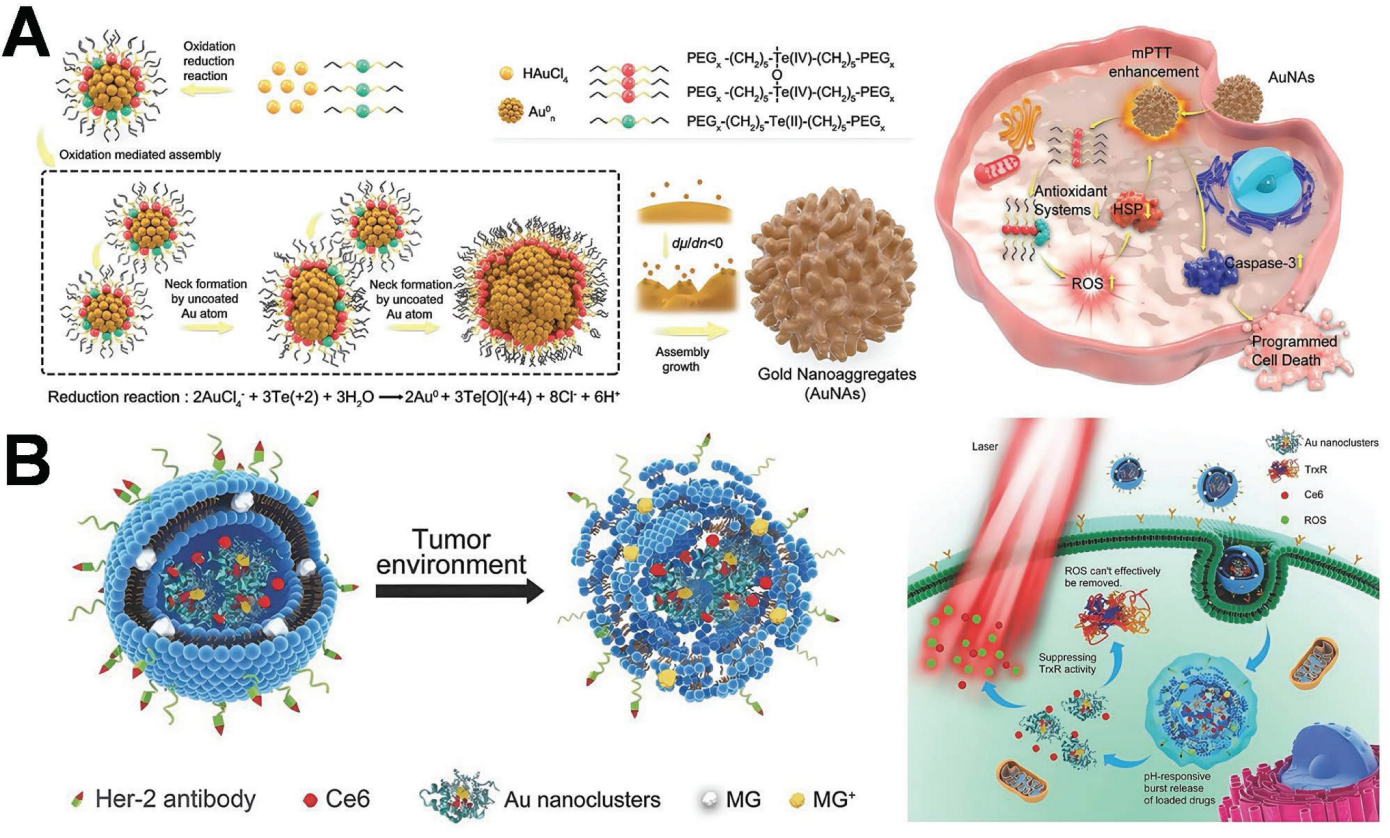
Schematic illustration of hot-spot empowered AuNPs for the therapy of breast cancer via PTT and PDT: (A) Schematic illustration of gold nanoaggregates enabling NIR LED-driven mild PTT and immune activation for effective breast cancer treatment. Adapted with permission from 149, Copyright 2025 Wiley. (B) Schematic illustration of gold nanoclusters and PSs Ce6 dual loaded spatiotemporal controllable liposomal nanocomposites to enhance tumor PDT effect by inhibiting TrxR. Adapted with permission from 152, Copyright 2017 Wiley.

PTT represents an innovative approach to tumor treatment, featuring non-invasiveness, minimal side effects, and enhanced targeting capabilities, thereby demonstrating considerable potential in promoting tumor therapy. Researchers have fabricated a multitude of photothermal agents demonstrating exceptional biocompatibility and photothermal conversion efficiencies, such as nanoparticles of precious metals (Au, Ag, platinum), metal chalcogenides, carbon derivatives (including carbon nanosheets, carbon nanotubes, graphene, and fullerenes), and polypyrrole (PPy) 146-148. Recently, researchers have successfully fabricated gold nanoaggregates (AuNAs) consisting of densely packed nanospheres. These nanospheres give rise to numerous hot-spot regions, significantly augmenting NIR absorption and enabling efficient photothermal conversion under low-power NIR LED irradiation. By leveraging this approach, complete tumor ablation was accomplished through sustained mild hyperthermia (approximately 45 °C), effectively circumventing collateral tissue damage. Significantly, this treatment induced robust immunogenic cell death (ICD), facilitating dendritic cell maturation and T cell activation. Consequently, photothermal therapy was integrated with immune enhancement to achieve effective breast cancer treatment (**Figure 13A**) 149. It is important to note that the current mainstream NIR-I band (750-900 nm) typically exhibits a penetration depth of no greater than 2.5 mm within breast tissue. Moreover, in high-density tumor tissue, the light intensity decays exponentially, this intrinsically limits the treatment of deep tumors 150.

PDT represents a minimally invasive tumor treatment modality with high selectivity and low side effects 151. Nevertheless, it often proves ineffective in the long-term control of tumors. One of the principal reasons lies in the fact that tumor cells establish certain protective mechanisms to assist them in coping with oxidative stress within the environment. The thioredoxin system in cancer constitutes an important antioxidant defense system. This study has developed a spatiotemporally controllable liposome nanocomposite co-loaded with gold nanoclusters and PSs **(Figure 13B)** 152. Gold nanoclusters disrupt the antioxidant defense system of tumor cells by specifically inhibiting thioredoxin reductase (TrxR), thereby significantly enhancing the ROS generation efficiency of the PS and its PDT effect. The synergistic action of gold nanoclusters and photosensitizers gives rise to an “oxidative stress storm”, achieving efficient elimination of breast cancer cells. However, the therapeutic efficacy of PDT is frequently influenced by the hypoxic microenvironment within the deep regions of tumors. Specifically, when the depth exceeds 3 mm, the local partial pressure of O₂ drops below 5 mmHg. This hypoxic condition can result in a 60-80% reduction in the production rate of ROS 153. In recent years, NIR-II responsive gold nanoclusters, such as those featuring a peak absorption at 1200 nm, have been demonstrated to substantially increase the penetration depth (up to 7.1 mm) and modulate the local oxygen supply. Consequently, this enhances the therapeutic effectiveness of deep PDT 154.

Hot-spot empowered AuNPs present distinct advantages in breast cancer therapy through PTT and PDT. AuNPs play a crucial role as hot-spot enhancers in PTT and PDT, alternatively functioning as carriers for PTA and photosensitizers to facilitate PTT and PDT therapy. The LSPR of AuNPs boosts light absorption and scattering, thereby enhancing the efficiency of photothermal and photodynamic effects 155-157. Nevertheless, monotherapy confronts challenges such as insufficient efficacy, damage to the surrounding healthy tissues, and the biosafety of photothermal agents, thereby restricting the clinical application of PTT and PDT. Consequently, it is of paramount importance to establish a platform based on multimodal integration therapy 158.

### 4.3 Multimodal integration therapy

Due to tumor heterogeneity and drug tolerance, monotherapy is unable to attain optimal therapeutic effects in the treatment of breast cancer. Researchers are actively seeking multiple cooperative treatments for breast cancer, with the aim of enhancing the efficacy and accuracy of tumor therapy by leveraging the complementary advantages of various therapeutic approaches. Extensive research suggests that integrative approaches encompassing multiple treatments, such as chemotherapy, radiation, PTT, PDT, and immunotherapy, yield favorable outcomes. Table 3 depicts the specific application of multimodal integration therapy employing hot-spot empowered AuNPs in breast cancer treatment.

Table 3 showcases several research publications in recent years, indicating that multimodal integration therapy employing hot-spot empowered AuNPs for breast cancer therapy constitutes a highly active field of study that has achieved remarkable results. Employing hot-spot empowered AuNPs, which demonstrate superior photothermal properties, multifunctional drug transport capabilities, and immune activation effects, provides a novel multimodal platform for the treatment of breast cancer 170. Nevertheless, it still keeps encountering obstacles such as biocompatibility, safety 171, treatment depth 172, individualized treatment design, and production cost 173. Future development ought to focus on enhancing the biocompatibility and targeting of AuNPs, resolving issues related to treatment depth and precise control, and minimizing costs to enhance the potential for clinical application.

### 4.4 Recent translational advances: from bench to bedside

With the advancement of nanomedicine, *in vivo* investigations of AuNPs in breast cancer treatment are progressively transitioning from mechanism validation to functional integration and translational applications. In recent years, numerous preclinical studies have employed animal models to comprehensively assess the efficacy and feasibility of AuNPs in combination therapy, targeted delivery, and material controllability. This has further augmented their application potential within the treatment framework.

The PTT-chemotherapy synergistic platform developed by Chen *et al.* achieved remarkable tumor inhibition (>85%) in the 4T1 breast cancer model. This was achieved by loading paclitaxel and activating it with NIR light. Additionally, the platform demonstrated excellent systemic tolerance. Notably, 180-day follow-up studies showed no evidence of hepatic fibrosis or renal dysfunction, attributed to the renal clearance of sub-10 nm PEGylated AuNPs 174. Ghaffarlou *et al.* developed an alginate-coated AuNPs system that enhanced the therapeutic response via the synergy between PTT therapy and low-dose radiotherapy. In animal experiments, no significant toxicity was detected, suggesting its effectiveness as a radiotherapy adjuvant strategy. Mass spectrometry analysis revealed<3% residual gold in liver/spleen at 60 days post-injection due to enzymatic degradation of the alginate matrix 175. Furthermore, the gold nanostars@PDA@Fe₃O₄ composite material developed by Li *et al.* showcases excellent targeted accumulation and thermal response capabilities *in vivo*. It possesses a stable thermal conversion efficiency, rendering it suitable for further integrated applications. The polydopamine coating reduced macrophage uptake by 65%, while Fe₃O₄ enabled real-time MRI monitoring of biodistribution 122. The gold nanoflower composite structure fabricated by Ersoy *et al.* via 3D printing demonstrates precisely controllable photothermal ablation capabilities and is anticipated to offer a novel construction platform for personalized treatment 176.

Notably, recent studies and reviews have emphasized long-term safety—addressing toxicity, immunogenicity, and organ accumulation. Strategies like PEGylation, PDA-coating, and enzyme-degradable matrices help mitigate immune response and facilitate clearance, supporting translational readiness. Continued optimization in particle size, surface chemistry, and architecture has improved stability and tumor microenvironment adaptability 123,177.

In summary, recent studies not only emphasize the development of enhanced therapeutic strategies but also reflect an increasing focus on clinical viability through biosafety evaluation and real-time tracking. These advancements provide a robust foundation for the precise, effective, and safe theranostic application of AuNPs in the treatment of breast cancer.

## 5. Application of hot-spot empowered AuNPs in the theranostics of breast cancer

The theranostics application in breast cancer represents a novel methodology that integrates the diagnosis and therapy of the disease. AuNPs hold considerable potential in the theranostic application for breast cancer, enabling precise detection and therapy through targeting, imaging characteristics, drug delivery, and PTT. AuNPs can precisely diagnose breast cancer and act as a therapeutic modality, demanding exceptionally fine structural properties. Consequently, optimizing the design of AuNPs to enhance their hot-spot region for the integrated diagnosis and therapy of breast cancer has remained a prominent issue for researchers in recent years.

Circulating tumor cells (CTCs) within blood vessels are the principal causes of cancer metastasis and mortality among cancer patients. The identification and elimination of CTCs are of vital significance for the early diagnosis and therapy of cancer. This study presented a novel nanolayer, namely AuNPs/gold nanorods@PDA, which is applied on Ω-shaped optical fiber (Ω-FO) for LSPR to facilitate the diagnostics of breast cancer cells and PTT 178. The Ω-shaped fiber LSPR configuration is capable of generating a multitude of hot-spot. A PDA nanolayer is deposited onto the uncoated optical fiber. The surface of the PDA nanolayer assimilates AuNRs and AuNPs, thereby giving rise to a hybrid nanolayer. Each hot-spot boosts photothermal conversion efficiency and enables more precise localization of the tumor region. The modified Ω-FO LSPR demonstrates remarkable refractive index sensitivity and a photothermal conversion efficiency of 37.59 (a.u/RIU). Upon modification with recognition molecules, the Ω-FO LSPR is transformed into a sensitive cellular sensor. Under NIR laser irradiation, the Ω-FO LSPR is capable of killing trapped tumor cells with an apoptosis/necrosis rate of 62.6%, showing minimal side effects on non-target cells. Notably, upon 808 nm laser irradiation (2.5 W/cm², for 5 min), the Ω-FO LSPR structure displayed a rapid temperature elevation from 25±1 °C to 69 °C (measured via an IR thermal camera), corresponding to a ΔT of~44°C. This value surpasses the thermal ablation threshold for tumor cells (42-45 °C) and is consistent with reported AuNP systems featuring η>30% 179. The simultaneous high refractive index sensitivity (37.59 a.u./RIU) and remarkable photothermal performance indicate that the plasmonic hot-spot within the AuNPs/gold nanorods@PDA hybrid architecture synergistically boost both LSPR sensing and photothermal conversion. This research integrated tumor cell detection and PTT, facilitating simultaneous diagnosis and therapy on a unified platform. The distinctive hot-spot architecture of AuNPs enables real-time monitoring of tumor alterations for customized therapy.

**Figure 14:**
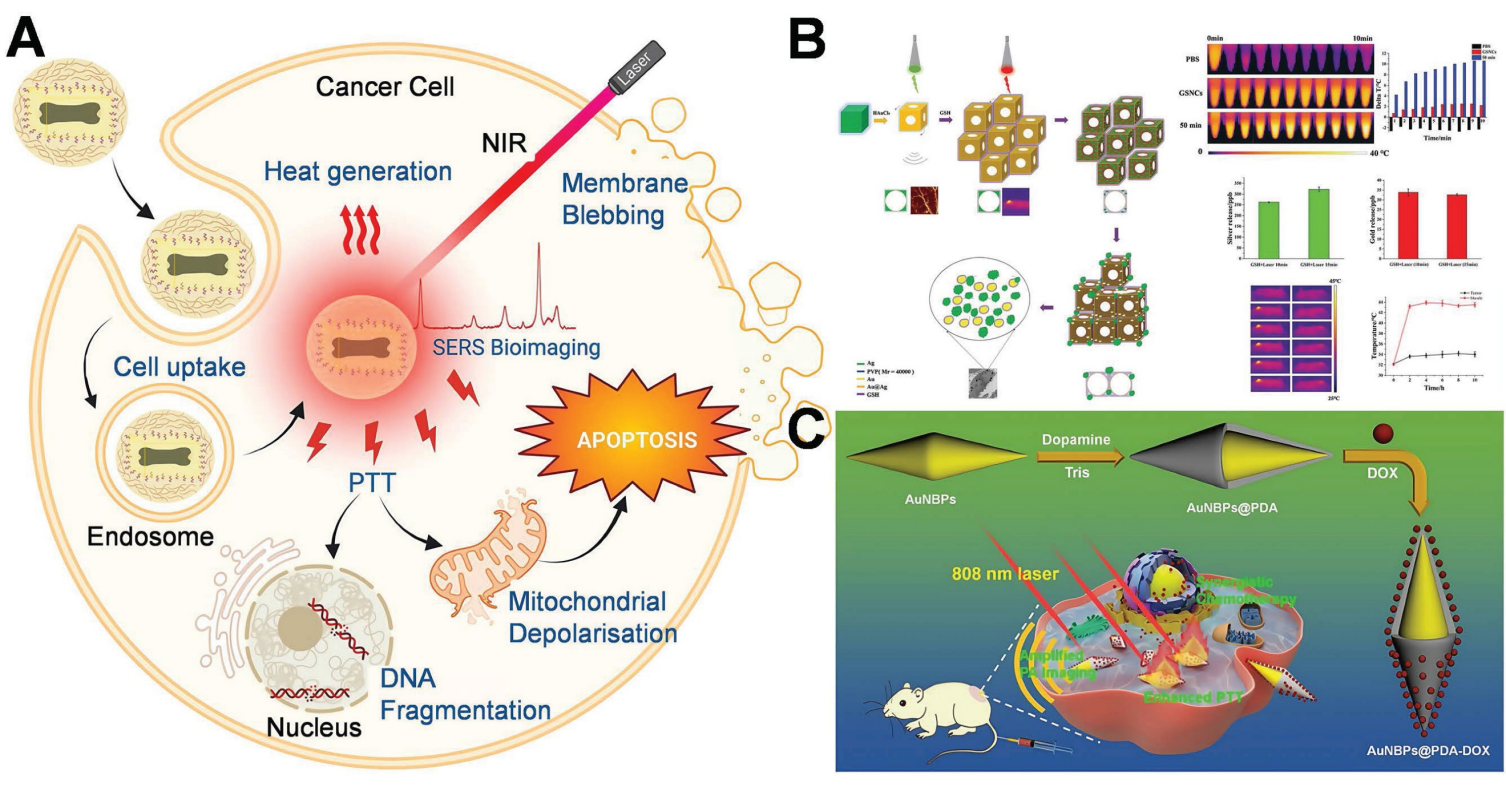
Schematic diagram of the application of hot-spot empowered AuNPs in the theranostics of breast cancer: (A) AuDB NRT nanostructures for breast cancer diagnosis and PTT. Adapted with permission from 180, Copyright 2024 American Chemical Society. (B) GSH-induced aggregation of Au-Ag nanocages (Au-Ag NCs) for *in vivo* PAM imaging and PTT. Adapted with permission from 181, Copyright 2019 Wiley. (C) Synthesis of AuNBPs@PDA-DOX nanoprobes, featuring PA signal amplification and improved photothermal conversion for integrated breast cancer detection and treatment. Adapted with permission from 182, Copyright 2020 American Chemical Society.

Amit Jaiswal developed a fascinating “gold dogbone nanorattles” (AuDB NRT) (**Figure 14A**) 180. This structure features two aspects: enhancing the LSPR of the nanoparticles and presenting a photothermal conversion efficiency of 35.29%. It demonstrates superior NIR absorption in contrast to conventional AuNPs. The structural design is crucial to the hot-spot phenomenon. AuDB NRT possesses a highly potent electromagnetic hot-spot capable of enhancing the signal of Raman reporter molecules, thereby making it an outstanding probe for SERS-based cancer cell bioimaging. NIR imaging facilitates the effective monitoring of the location and size of breast cancer tumors with considerable penetration. Cationic dextrin nanoparticles encapsulate AuDB NRT, thereby enhancing its stability, biocompatibility, and diagnostic targeting. Upon exposure to a near-infrared laser, AuDB NRT generates heat, the maximum temperature increase amounts to 38°C, effectively eliminating tumor cells within a shortened time frame.

In another investigation, researchers utilized GSH for the synthesis of partially hollow gold-silver nanocage (GSNC) structures (**Figure 14B**) 181. This hollow configuration will modify the surface morphology of the nanostructure, especially in the holes or recessed areas of the nanocage, which can promptly generate hot-spot and significantly enhance the photothermal effect. The surface modification gives rise to significant absorption peaks within the visible to near-infrared spectrum, facilitating its application as a contrast agent for optical imaging and thereby enabling photoacoustic microscope imaging for real-time breast cancer diagnosis. The injection of GSNCs into the tumor region and subsequent exposure to NIR irradiation allows the GSNCs to absorb NIR and convert it into thermal energy. The accumulation within the tumor tissue leads to a significant rise in temperature at the tumor site, inducing thermal damage to the cancer cells and ultimately culminating in cell apoptosis or death. In a comparable vein, Zeng *et al.* fabricated polydopamine (PDA)-coated gold nano bipyramids (AuNBPs@PDA) (**Figure 14C**) 182 to augment the photoacoustic signal and enhance photothermal conversion under low-dose laser irradiation. In this study, through the employment of PTT, a synergistic treatment paradigm integrating PTT and chemotherapy is put forward. The PDA coating has the ability to encapsulate chemotherapeutic agents and facilitate their release within tumor microtissues.

The design and investigation of gold nanostructures for the theranostics of breast cancer through the utilization of hot-spot enhancement are still in the research and development stage. Numerous research investigations persistently explore the integration of AuNPs with conventional modalities, including radiotherapy, chemotherapy, and gene therapy, in order to achieve precision treatment. Recent studies have emerged as researchers have increasingly focused on the application of integrated diagnosis and therapy, thereby enhancing efficiency and reducing the cost of research to achieve an integrated approach for the diagnosis and therapy of breast cancer, the hot-spot that enhance the effect of AuNPs can be effectively incorporated into these processes. The synthesis and design of AuNPs demand extremely stringent conditions. The dimensions, morphology, surface characteristics, and other attributes of AuNPs have to be meticulously controlled to attain the optimal hot-spot amplification effect. Integrating the enhancing effect of the hot-spot with other diagnostic or therapeutic approaches poses a significant challenge 183. Despite the promising results of AuNPs *in vitro* studies, their biocompatibility, safety, and *in vivo* performance need to be assessed prior to their translation into clinical applications 176. Consequently, the design of gold nanostructures that could successfully enhance diagnostic and therapeutic outcomes without adverse effects on the human body poses a significant challenge and a key focus for future research.

## 6. Summary and future perspectives

Currently, the AuNPs technology centred on the enhancement of hot-spot is evolving from the preparation of effective hot-spot to precise regulation and eventually achieving efficient utilization of these hot-spot. This review explores the hot-spot that enhance the process of AuNPs, which are subsequently employed in the diagnosis and therapy of breast cancer. AuNPs utilize optical diagnostic techniques, such as LSPR and SERS, in conjunction with electrochemical sensing approaches to facilitate highly sensitive and precise detection of breast cancer, thereby enhancing the accuracy of early diagnosis. The targeted drug delivery, PTT, PDT, and multimodal integration therapy of AuNPs have manifested remarkable therapeutic efficacy, instilling new life and confidence into personalized medicine. Furthermore, the novel application of AuNPs in the theranostics of breast cancer has forged a robust integration and seamless connection between diagnosis and treatment, thereby markedly promoting the sustainable development of precision medicine.

Despite the considerable potential of AuNPs in demonstrating hot-spot enhancement for the diagnosis, therapy, and theranostics of breast cancer, they confront numerous obstacles in practical application. Control and stability of hot-spot. Although contemporary design and synthesis techniques can partially regulate the distribution of hot-spot in AuNPs, precisely controlling the structure of these hot-spot still poses a challenging problem. Moreover, in a complex biological environment, the hot-spot structure may lose its functionality due to particle aggregation, degradation, and other factors, significantly reducing the stability of its enhancing effect.

Biocompatibility and safety. While AuNPs exhibit excellent chemical stability and biocompatibility, their clinical translation faces three key safety challenges. (1) Long-term toxicity—accumulation in the reticuloendothelial system (particularly the liver and spleen) may trigger chronic inflammation at therapeutic doses. (2) Immunogenicity—surface modifications must be carefully engineered to minimize antibody production and complement activation. (3) Off-target accumulation—precise control of biodistribution and enhanced clearance strategies are critical to reduce retention in non-target organs. Addressing these issues through rational material design and comprehensive *in vivo* safety evaluation is essential to enable clinical application.

Balancing in the design of theranostics. To meet the requirements of theranostics, AuNPs often need the integration of multiple functionalities, such as optical capabilities, drug delivery, thermal therapy, and imaging. This multifunctional design imposes extremely strict constraints on gold nanostructures. Precise control of the size, shape, surface characteristics, and other factors of AuNPs is crucial to achieve the optimal hot-spot enhancement effect. Integrating the hot-spot enhancing effect with other imaging or therapy approaches poses a significant challenge. Therefore, achieving a balance between the multifunctional design and the hot-spot performance is an issue that demands in-depth investigation.

The implementation of customized therapy. Individual variables, such as tumor type, molecular characteristics, and treatment response, can significantly affect the efficacy of AuNPs in the diagnosis and treatment of breast cancer. Nevertheless, the AuNPs technology employing hot-spot enhancement holds considerable potential for further exploration in personalized treatment, including the design of particle performance customized to individual patient traits and the improvement of targeting accuracy, among others. Consequently, designing personalized hot-spot empowered AuNP solutions tailored to patients' specific conditions is a significant research direction for the future.

Synergistic integration of LSPR and SERS for enhanced theranostics. The complementary strengths of LSPR and SERS present a promising opportunity to advance AuNP-based diagnostics and therapeutics for breast cancer. LSPR enables real-time, label-free detection of biomolecular interactions with high sensitivity to the local environment, while SERS offers molecular fingerprinting at ultralow concentrations. Integrating both modalities on a single AuNP platform allows for multiplexed biomarker monitoring (via LSPR shifts) and spatially resolved imaging (via SERS), paving the way for more precise, targeted intervention. SERS-identified tumor hot-spot could guide LSPR-triggered photothermal therapy, establishing a closed-loop theranostic strategy. Preliminary *in vitro* studies—such as dual-mode exosome detection and HER2 analysis—have demonstrated this synergy. Nevertheless, challenges remain in adapting these systems to complex *in vivo* conditions. Future efforts should focus on designing multifunctional AuNPs that retain dual-modal activity in physiological environments and leveraging AI-driven approaches to correlate LSPR and SERS outputs for individualized treatment optimization.

In the face of these obstacles, several disciplines need to undertake coordinated endeavors, encompassing materials science, nanomedicine, and biomedicine. Through design optimization and the integration of novel technologies, the AuNPs technology exploiting hot-spot enhancement will progress towards practical application. This will boost the diagnostic and therapeutic efficacy of breast cancer, while providing crucial reference experiences and exemplars for promoting precision medicine in other cancer types.

## Figures and Tables

**Table 1:** A quantitative comparison of the structural parameters of AuNPs and the enhancement effect of hot-spot.

AuNP structure	Geometric characteristics	Hot-spot distribution	Enhancement factor (EF)	Relative SERS intensity	Representative application	Refer
Monomer (spherical)	Diameter~60 nm	Isolated particle, no hot-spot	~10³	Baseline (1×)	Basic sensing with low sensitivity	22,35
Dimer	Interparticle gap ~1-2 nm	Single interparticle hot-spot	10⁴-10⁶	~16×compared to monomer	Single-molecule detection (e.g., DNA mutations)	35
Linear trimer	Three particles linearly arranged, 2 nm gap	Two interparticle hot-spot	10⁶-10⁷	~87×compared to monomer	High-sensitivity biomarker detection (e.g., HER2)	35
Triangular trimer	Symmetrical triangular assembly, 2 nm gap	Three interparticle hot-spot	10⁷-10⁸	~120×compared to monomer	Multi hot-spot enhancement for complex samples	37
Heptamer (CN=7)	Pentagonal bipyramidal structure, <2 nm gap	Coupled hot-spot cluster (>10 sites)	10⁸-10⁹	~4000×compared to monomer	Ultrasensitive exosome detection	37
Nanoflower (8 petals)	Multi-branched, tip radius~5 nm	Dense hot-spot between petals	10⁷-10⁸	Broadband enhancement	Wide-spectrum plasmonic sensing and imaging	38

**Table 2:** Comparison of representative gold nanostructures for hot-spot applications.

AuNP structure	Synthesis method	Fabrication ease	Advantages	Disadvantages	Stability	Application areas	Refer
Gold dimers	DNA-directed assembly	Medium (depends on connection efficiency)	SERS signal 87× higher than monomer	Prone to aggregation in high-salt conditions	Inactive in serum without surface modification	Single-molecule detection	35
Gold nanotrimers	Ligand-exchange driven self-assembly	Relatively difficult (sensitive to solvent polarity)	87×SERS enhancement in triangular configuration	Solvent polarity affects configuration	Stable in organic media	Multi hot-spot enhancement	35
Gold nanocubes	Colloidal chemical reduction	Easy (simple morphology control)	Uniform shape, facile functionalization	Low EF as monomer; dimerization required	Dispersion dependent on ligands	Basic sensing platform	22
Gold nanostars	Seed-mediated growth	Relatively difficult (precise control of branch growth is required)	Multiple hot-spot, broadband absorption (545-725 nm)	Highly dependent on tip curvature	PEG modification required in serum to prevent aggregation	PTT	22
Multi-petal aggregates	Spark discharge with electrostatic focusing	Relatively difficult (The number of petals needs controlling)	Broadband enhancement across visible spectrum	Structural heterogeneity	Signal attenuation at ionic strength>150 mM	Plasma-based devices	38
DNA-templated assemblies	DNA origami	Extremely difficult (time-consuming>24 h)	Programmable positioning at molecular scale	Synthesis time>24 h	Susceptible to nuclease degradation	Ultra-sensitive diagnosis	52
Nanoflowers	Peptide-mediated anisotropic growth	Relatively difficult (complicated branch control)	High surface roughness	Uneven branch length	pH-sensitive (structural changes under acidic conditions)	Chiral sensing	60
Core-shell structures	Template-assisted (SiO₂ coating)	Medium (the shell thickness needs to be controlled)	Protects core from environment	EF drops if shell>10 nm	Stable sub-5 nm gap in physiological conditions	Biosensing	67

**Table 3:** Application of AuNPs-based multimodal integration therapy in breast cancer.

AuNP structure	Combination therapy	Key geometric parameters	Enhancement factor (EF)/Metric	Tumor regression (%)	Therapeutic window	Refer
Gold nanocages with cancer cell membrane	Chemotherapy +PTT	Cage wall thickness~5 nm; membrane coating	η:37.6% (808 nm, 1.5 W/cm²); pH-triggered release	Tumor volume reduced by 82.2% (day 14); metastasis suppressed	~7 days (one cycle)	159
Janus-type AuNPs (Octopus)	Chemotherapy +PTT	Aspect ratio~3.8; core-satellite morphology	NIR absorption ↑; targeting enhanced by anisotropic design	Tumor volume inhibition: 60%; targeting accumulation ↑2.5×	___	160
EV-guided gold nanopopcorn	Chemotherapy +PTT	Diameter~80 nm; spike-shaped	η:39%; ΔT=50.2 °C (808 nm, 1.5 W/cm²)	Tumor volume reduction: 89%; pH-responsive drug release	~6 days	161
Liposome-coated gold nanostars@NMOF	Chemotherapy +PTT+PDT	Core-shell; shell thickness~10 nm	pH-sensitive drug release 85% at pH 5.0	Tumor-free survival>60 days	>60 days	162
Gold nanostars@MSN	Chemotherapy +PTT	Star-shaped core, ~100 nm; mesoporous silica shell	η:~49%; dual-release at low pH and high temperature	Tumor growth inhibition rate: ~91%	___	163
Ce6-conjugated PDA-gold nanostars	PTT+PDT	Gold nanostars with tip radius <5 nm; PDA shell	ROS ↑4.8×; ΔT: 44°C (2 W/cm²); strong PA signal	Complete remission in 67% of mice; lung metastasis reduced	~6 days	164
Gold nanoclusters@MOF	PDT +Chemotherapy	MOF pore~2.1 nm; gold nanocages core	ROS ↑3.7×; pH-sensitive drug release	Tumor weight reduced by 72%	~7 days	165
Aggregated AuNPs-PS conjugates	PTT+PDT	Aggregated PS-AuNPs (<100 nm)	Enhanced photoactivity under NIR	Tumor growth suppressed under sub-threshold irradiation	___	166
Au@PDA-PEG-MTX	Chemotherapy +PTT	Core~45 nm; PDA layer; PEG-MTX conjugation	η:35%; drug release: 78% (pH 5.0)	Tumor inhibition rate: 84%; targeting accumulation ↑3.2×	~6 days	167
HER2-gold nanostars (CDK-assisted)	Chemotherapy +PTT +Immunotherapy	Size~65 nm; HER2-mAb density: 12/μm²	ΔT: 48°C at 2 W/cm²; CD8⁺ T-cell infiltration ↑3.5×	Lung metastasis suppression: 93%; antigen binding rate: 89%	~7 days	168
Gold nanocages co-assembled with spinacia oleracea extract	PTT+PDT	Diameter~70 ± 10 nm	ROS generation ↑ under 808 nm; photothermal conversion under NIR confirmed	___	___	169

η: photothermal conversion efficiency; ΔT: local temperature increase; PA: photoacoustic signal; MOF: metal-organic framework; PEG-MTX: polyethylene glycol-methotrexate; PDA: polydopamine; MSN: mesoporous silica nanoparticles.
